# Sleep prevents brain phosphoproteome disruption to safeguard survival

**DOI:** 10.1038/s41421-025-00809-w

**Published:** 2025-06-24

**Authors:** Jing Ma, Juhang Liu, Yu Li, Yikui Zhao, Yu Tian, Bing Hu, Kaiyue Yan, Ying Li, Kaihang Ding, Xiangyu Wang, Huiwen Tian, Wen Si, Ketong Liu, Huiran Zhang, Chongchong Zhao, Guangfu Wang, Zhiqiang Wang

**Affiliations:** The HIT Center for Life Sciences, School of Life Science and Technology, Harbin Institute of Technology, Harbin, Heilongjiang China

**Keywords:** Proteomics, Bioinformatics, Proteomic analysis, Phosphorylation

## Abstract

Prolonged sleep deprivation (Pr-SD) causes death in many species. While various mechanisms related to sleep regulation or this fatal consequence of sleep loss have been identified, the core molecular basis linking Pr-SD-induced lethality and sleep homeostasis remains unknown in mammals. A critical “point of no return (PONE)” status in Pr-SD subjects is highlighted in classic research, and characterizing PONE status could help uncover this mystery. Using a Pr-SD model and a reliable PONE status prediction method, we show that mice in PONE exhibit an inability to enter natural sleep, and significant disruptions in brain phosphoproteome, independent of deprivation time but closely linked to PONE status. Brain kinase or phosphatase dysfunction influences PONE status development and leads to corresponding sleep aberration concurrently. Daily 80-min recovery sleep significantly delays PONE onset and restores brain phosphoproteome. The harmful effects of excessive kinase activity on PONE development can be eliminated by combining recovery sleep and compensatory phosphatase expression. We conclude that sleep is crucial for maintaining brain phosphoproteome homeostasis, whose disruption may impact both Pr-SD-induced lethality and sleep regulation.

## Introduction

Sleep is an indispensable behavior conserved across all animal species^1–4^. Over the past decades, numerous factors that regulate the quantity or quality of sleep have been reported, ranging from some circadian genes^5^ and sleep-related circuits^6^ to specific kinase signaling pathways^7^ and neurotransmitters^8^. As a homeostatic system, it has been posited that the core molecular basis of sleep should govern both its regulation and its primary function^9,10^. By examining the effects of sleep deprivation on varying aspects, many sleep-dependent functions have been proposed, including cognition, metabolite clearance, metabolism and immunity^10–13^. Studies on prolonged sleep deprivation (Pr-SD) have also been conducted to dissect the primary function of sleep^14^, uncovering several mechanisms underlying Pr-SD-related lethality, such as the accumulation of reactive oxygen species (ROS) in the gut of flies^15^, and the cytokine-storm-like syndrome in the peripheral system of mice^16^.

Despite the fact that sleep plays a profoundly significant role in the central nervous system, up to now, no morphological or functional changes in the brain have been identified as mechanisms underlying Pr-SD-related lethality^14–17^. As a result, the elusive lethal mechanism of Pr-SD and the core molecular basis of sleep homeostatic regulation are further shrouded in mystery.

Current methods for studying Pr-SD and its lethality remain limited. Gentle handling, a common approach, is ineffective for extended deprivation^15,16^. The disk-over-water (DOW) method, while useful in rats, is hindered by scarce genetic tools for this species^14^. In mice, DOW is impractical due to restricted mobility post-electrode implantation caused by their smaller size. Improved models are needed to address these species-specific challenges.

In DOW of rat studies, a “point of no return (PONE)” status has been observed, characterized by the irreversible nature of subjects’ death even when the deprivation procedure was terminated^18,19^. Accurately identifying mice in the PONE status and characterizing the profiles of this enigmatic status may contribute to understanding the mechanisms underlying Pr-SD-induced lethality. Our PONE status indexing analysis, along with proteomic, bioinformatic and genetic studies, demonstrated that brain phosphoproteome balance is important for both sleep regulation and Pr-SD-induced organismal death.

## Results

### A Pr-SD sleep deprivation mouse model

**Fig. 1:**
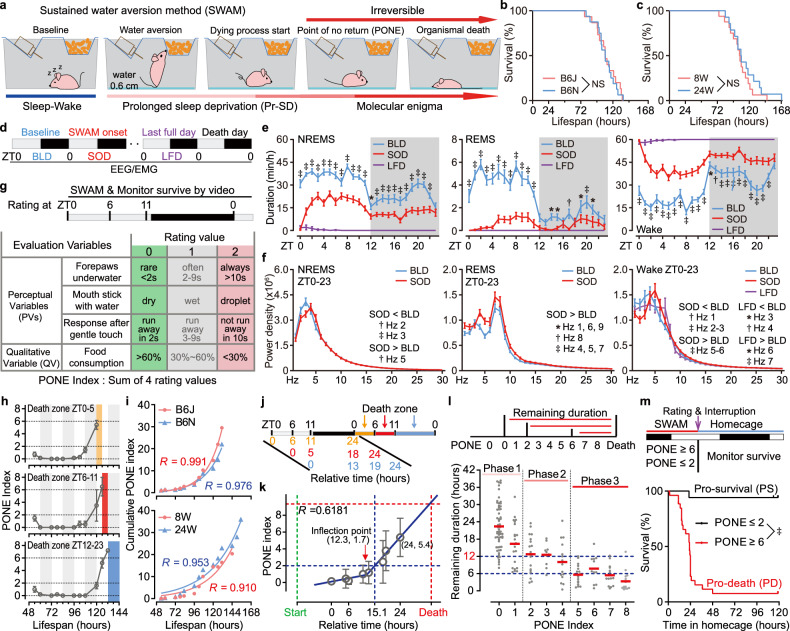
Evaluation of the PONE status during Pr-SD. **a** Schematic diagram of SWAM. **b**, **c** Survival analysis of SWAM-treated mice: C57BL/6 J (B6J, *n* = 15) vs C57BL/6 N (B6N, *n* = 16) mice (**b**), B6J cohorts aged 8 weeks (8 W, *n* = 16) vs 24 weeks (24 W, *n* = 14) (**c**). **d** Schematic of SWAM-treated mice undergoing continuous EEG/EMG recording. Baseline day (BLD), SWAM onset day (SOD), last full day (LFD). **e**, **f** Analysis of duration (**e**) and absolute EEG power spectra (**f**) of NREMS, REMS and wake states of B6J mice (*n* = 18 per group) on BLD, SOD and LFD. **g** Schematic of PONE status evaluation and PONE index calculation. **h** Representative PONE index development for the mice died on the 6th SWAM day during ZT0–5 (yellow, *n* = 7), ZT6-11 (red, *n* = 2) or ZT12-23 (blue, *n* = 5). **i** Cumulative mean PONE index value of B6J (*n* = 15) and B6N (*n* = 15) mice (top), and B6J mice aged 8 W (*n* = 14) and 24 W (*n* = 17) (bottom). **j**, **k** Relative timeline (**j**) and simulating analysis by segmental linear regression (**k**) for the last four PONE index rating in three scenarios. **l** A plotting of the PONE index values against the remaining survival duration from each rating time to the actual death point (*n* = 60). Red line represents mean survival. **m** Survival analysis of SWAM interruption test for mice with PONE index ≤ 2 (*n* = 26) and PONE index ≥ 6 (*n* = 17). Data are mean ± s.e.m. Log-rank (Mantel-Cox) test (**b**, **c**, **m**); two-way ANOVA with Dunnett’s test (**e**, **f** right); two-way ANOVA with Sidak’s test (**f**); Exponential growth equation Least squares fit (**i**). *P < 0.05; †*P* < 0.01; ‡*P* < 0.001; NS, not significant, *P* > 0.05. *n* refers to the number of biological replicates.

A complete and long-lasting sleep deprivation method is of crucial importance for sleep studies. However, the attempt to prolong acute sleep deprivation by means of traditional approaches, such as gentle handling, is hampered by the phenomenon of rebound sleep and the occurrence of local neuron “offline” during homeostatic sleep regulation^20,21^. Inspired by the seminal DOW studies, which demonstrated that rodents wouldn’t sleep in water^22,23^, we adopted a sustained water aversion method (SWAM) to carry out Pr-SD in mice. This method enables efficient performance of more genetic and biochemical studies. Mice were individually housed in rectangular cages filled with ~0.6 cm of water, and accurate time of death was defined when a mouse remained immobile for 5 min (Fig. 1a). Indeed, we observed a median survival duration of ~108.4 h in SWAM-exposed mice, consistent across different mouse substrains (C57BL/6 J, B6J and C57BL/6 N, B6N) and ages (8 weeks and 24 weeks) (Fig. 1b, c).

Furthermore, we conducted a concurrent electroencephalogram and electromyogram (EEG/EMG) analysis during the SWAM test, comparing the sleep-wake architecture among baseline day (BLD, the day before the SWAM test), SWAM onset day (SOD) and the last full day (LFD, which refers to the last complete Zeitgeber time (ZT) 0–23 before the death event) (Fig. 1d and Supplementary Fig. S1a). Sleep duration decreased gradually throughout the SWAM process, from 768.2 min during BLD to 361.5 min on SOD, and nearly disappeared on LFD (6.3 min), as did the durations of non-rapid eye movement sleep (NREMS) and rapid eye movement sleep (REMS) episodes (Fig. 1e and Supplementary Fig. S1b). Mice experienced about 45 h of complete sleep deprivation before death, which was independent of survival duration (Supplementary Fig. S1c). Compared to the absolute EEG power of BLD, we observed a significant suppression in NREMS delta power on SOD (2–3 Hz), wake delta power of SOD (1–3 Hz) and LFD (3–4 Hz) (Fig. 1f). These observations indicate that SWAM causes a significant reduction in both sleep quantity and quality, which underlies inescapable death.

### A method to predict PONE status precisely

Behavioral debilities have been utilized to assess the physiological conditions of rats subjected to DOW sleep deprivation^19,23^. In the SWAM model, we observed similar Pr-SD effects as seen in DOW-treated rats^17,19,23^. To predict PONE status, we employed a combination of 4 behavioral variables to monitor the subjects (Fig. 1g). More specifically, we deployed 1 Qualitative Variable (QV) and 3 Perceptual Variables (PVs), calculating the sum of 4 rating values to assess mouse status at 3 fixed ZT times (ZT0, 6 and 11) each day (Fig. 1g). To minimize subjective discrepancies as much as possible, we defined 2 boundary states which represent “pretty healthy” or “extremely feeble” (scored as 0 or 2) for each PV, and introduced an intermediate state (scored as 1) including all ambiguous states that could not be confidently categorized into either of the 2 boundary states (Fig. 1g). Applying this evaluation method, we found a similar PONE index development pattern across mice with varying survival times, characterized by a long-lasting initial stationary phase followed by a rapid growth phase (Fig. 1h). Mice from different substrains or ages also exhibited a consistent PONE index development curve after mitigating the influence of varying survival duration by computing the growth curve of cumulative PONE index (Fig. 1i and Supplementary Table S1). These findings underscore the reproducibility of our scoring system, and consistency of PONE index development.

Next, to further understand the PONE index development, we performed a data simulation analysis to quantify the velocity shift of PONE index (Fig. 1j). We recorded a series of PONE index values for every subject. However, we found that the final one was located at different ZT time points due to the death events that occurred in different time periods (Fig. 1h). To analyze the time-dependent development of the PONE index accurately, we employed only the last four PONE index values for each subject and aligned them within a relative 24-h timeline (Fig. 1j). The simulation analysis identified an inflection point at 1.7, after which PONE index quickly developed to the final value of ~5.4 before death (Fig. 1k). This suggests a transition point likely occurs at a PONE index of 2 in reality. To examine whether the PONE index reflects the probability of death, we plotted the PONE index against the remaining survival duration from each rating time to the actual death time. Results showed that mean survival time decreased progressively from ~22.4 h (PONE index = 0) to ~12 h (PONE index = 2–4), and further declined to ~6 h once the PONE index exceeded 5 (Fig. 1l and Supplementary Table S1). To test whether we could capture the PONE status successfully, we returned mice to their homecages after several days of sleep deprivation and PONE index rating (Fig. 1a, m). We carried out this SWAM interruption test in a paired manner and recorded the mortality of 2 groups: PONE index ≤ 2 group (whose members had not yet entered the rapid growth period) and PONE index ≥ 6 group (whose members theoretically experienced at least another 6 h of SWAM after crossing the inflection point) (Fig. 1k–m and Supplementary Discussions 1 and 2). Interestingly, although both groups of mice experienced the same SWAM duration, the mortality rates differed significantly — 5.2% vs 92.7% (Fig. 1m and Supplementary Fig. S1d). This discrepancy suggests that mice with PONE index ≥ 6 have entered an irreversible PONE status, jointly representing the two distinct poles of status alongside the other group (PONE index ≤ 2) (Fig. 1a, m). Accordingly, we termed two groups pro-survival (PS) and pro-death (PD) to reflect their potential outcomes of survival or mortality.

Together with the simulation data (Fig. 1k), these results suggest the Pr-SD-induced process may undergo three distinct phases: 1) an initial longstanding resistant phase (from start to PONE index = 2) that an intrinsic protective mechanism may support the subjects with normal behavioral responses; 2) a quick developmental phase (PONE index from 2 to 5/6) that the protective mechanism is rapidly out of action and the subjects showed abnormal behaviors; 3) the final dying phase (PONE index from 5/6 to death) that lethal factors build up and the process becomes irreversible (Fig. 1a, h, k and m).

### Pr-SD causes functional decline of brain electrical activity coupled with PONE status

After successfully capturing the point of no return status with the SWAM method and the PONE index system, we tried to dissect the causal factors related to the emergence of the PONE status. First, we offered water-soaked sponges to mice undergoing SWAM as an effort to replicate the water stimulus, and to allow for free-will sleep (Supplementary Fig. S1e). No mortality or PONE-like status was observed during the sponge SWAM, and all mice were released after 20 days (Supplementary Fig. S1e). The body weight of mice exhibited a mild decrease, and there was no difference between PS and PD mice, which could be considered as a side effect of sleep deprivation (Supplementary Fig. S1f). We further uncovered that the survival duration exhibited no correlation with either the subjects’ body weight or basal food intake (Supplementary Fig. S1g, h). Likewise, neither the illumination period nor melatonin (which is an antioxidant) had an impact on mortality (Supplementary Fig. S1i, j). Additionally, the daily food consumption during SWAM exhibited a typical circadian pattern, suggesting that the circadian system remained intact and that ROS was not a causal factor for mortality (Supplementary Fig. S1i–k). Moreover, the blood–brain barrier of PD mice did not display abnormal changes as observed in other sleep deprivation methods^16^ (Supplementary Fig. S1l). Collectively, these combined findings indicate that these potential confounding factors are not involved in the process of mortality^14^.

**Fig. 2:**
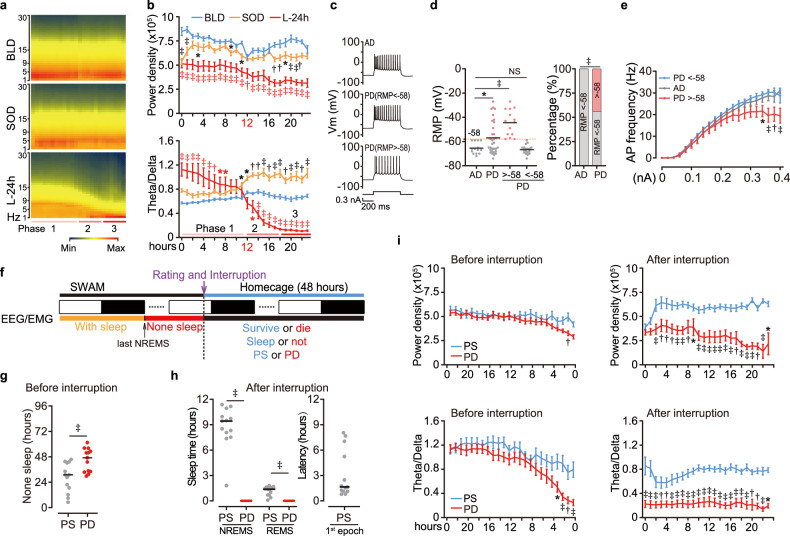
Functional changes in the brains of mice with PONE status. **a**, **b** Heat map plot of absolute EEG power (**a**), mean absolute EEG power (**b,** top), and theta/delta ratio (**b** bottom) of BLD, SOD and the last 24 h (L-24h) before the actual death time point (*n* = 15 per group). **c** Representative trace of action potentials of motor cortex layer 5 pyramidal neurons from AD mice (top, *n* = 14), PD mice with normal resting membrane potential (RMP) (middle, *n* = 18), and PD mice with RMP > -58 mV (bottom, *n* = 14). **d**, **e** RMP (**d** *left*), percentage of neurons with RMP > -58 mV (**d** *right*) and action potential frequency (**e**) were calculated for AD or PD neurons. **f** The paradigm of SWAM interruption test and relating parameter definitions. **g** Time within none-sleep period of PS and PD mice (*n* = 13 per group). **h** Sleep time (left) and sleep latency (right) within 24 h after SWAM interruption of PS (*n* = 13) and PD (*n* = 14) mice. **i** Mean absolute EEG power (top) and theta/delta ratio (bottom) during 24-h periods before (left) and after (right) SWAM interruption in PS (*n* = 13) and PD (*n* = 14) mice. Data are mean ± s.e.m. Two-way ANOVA with Dunnett’s test (**b**, **e**); one-way ANOVA with Dunnett’s test (**d** left); two-sided Chi-square (**d** right); unpaired *t*-test (**g**, **h**); two-way ANOVA with Sidak’s test (**i**). *P < 0.05; †*P* < 0.01; ‡*P* < 0.001; NS, not significant, *P* > 0.05. *n* refers to the number of biological replicates.

Next, we focused on the changes in brain electrical activity, and examined the absolute EEG power of the last 24 h (L-24h) before the time point of actual death and compared it with the BLD and SOD (Fig. 2a and Supplementary Fig. S2a–g). Consistent with the reduction in both sleep quantity and quality (Fig. 1e, f), the total absolute EEG power, which is mainly dominated by NREMS delta band in BLD, showed a significant decrease in SOD and L-24h (Fig. 2a, b). The ratio of theta (5–8 Hz) vs delta power band was elevated in SOD and first half of L-24h (Fig. 2b). Notably, the theta/delta ratio in L-24h exhibited a plunge at about 12 h before death, and reached its lowest point 6 h prior to death, as well as theta, alpha and beta power bands (Fig. 2b and Supplementary Fig. S2e–g). This pattern aligned closely with the three-phase development of the PONE index (Figs. 1l, 2b). These results indicated that the potential changes in neuron firing pattern in the brain. Therefore, we measured the basic electrophysiological properties of layer 5 pyramidal neurons in the primary motor cortex from either ad libitum sleep (AD) or PD mice using current clamp recordings (Fig. 2c). Approximately 44% of PD neurons exhibited significantly higher resting membrane potential (RMP > -58 mV) and decreased firing frequencies (Fig. 2d, e). To demonstrate the differences between mice with different survival outcomes, we further examined the EEG data of SWAM interruption test (Fig. 2f). The none-sleep period between the last NREMS epoch and interruption point showed significant difference in PS and PD mice (Fig. 2g). Notably, only PS mice entered normal sleep eventually after returning to the homecage, regardless of varying latency duration (Fig. 2h). Besides, the absolute EEG power and the theta/delta ratio of PD subjects were significantly lower than those in PS mice, and could not recover even after SWAM discontinuation (Fig. 2i). Together, these results strongly indicated a decline in brain electrical activity during SWAM process and PONE status.

### The phosphoproteomic disruption of brain in PONE status

**Fig. 3:**
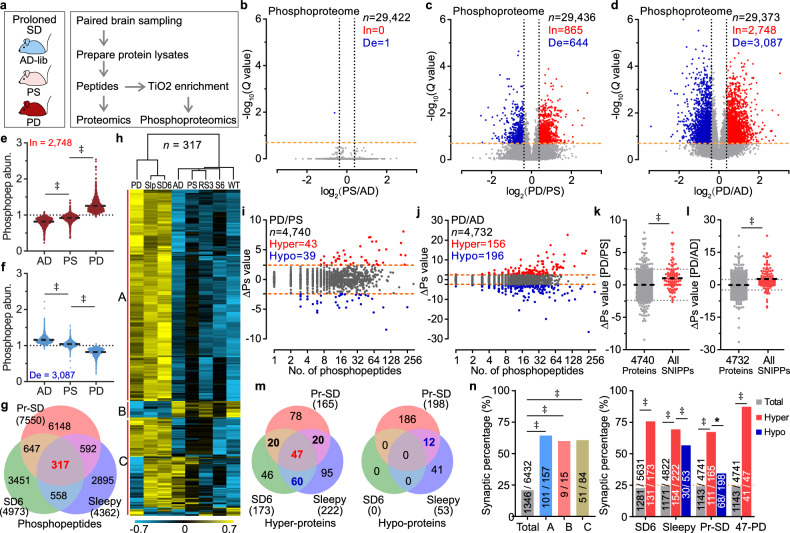
Disturbance of brain phosphoproteome during PONE status. **a** Experimental design for quantitative proteomic and phosphoproteomic studies of Pr-SD model. **b**–**d** Volcano plots displaying changed phosphopeptides in PS/AD (**b**), PD/PS (**c**) and PD/AD (**d**) groups. In, increased [log_2_(ratio) > 0.3]; De, decreased [log_2_(ratio) < −0.3]. Multiple unpaired *t*-test (*P* value) followed by false discovery rate (FDR) (*Q* value) analysis. **e,** **f** Analysis of mean abundance of increased (**e**) or decreased (**f**) phosphopeptides in PD/AD comparison (**d**). **g**, **h** Venn diagram (**g**), hierarchical cluster and classification analysis (**h**) of 317 significantly changed (*Q* < 0.2) phosphopeptides among three groups with total number in parentheses (**g**). Pr-SD represents all significantly changed peptides in PD/PS or PD/AD groups. **i**–**l** Global ΔPs analysis of phosphoproteins in PD/PS (**i**) and PD/AD (**j**) groups, and quantitative ΔPs analysis of SNIPPs in PD/PS (**k**) and PD/AD (**l**) comparisons. Dotted lines represent ΔPs = ±2.4. Numbers of total, hyperphosphorylated (Hyper) and hypophosphorylated (Hypo) proteins are shown. **m** Venn diagram showing overlaps of Hyper-phosphoproteins (ΔPs > 2.4, left) and Hypo phosphoproteins (ΔPs < −2.4, right) among three models. **n** Synaptic percentage analysis for class A–C phosphoproteins (left), Hyper-, Hypo- and PD-SNIPPs phosphoproteins (right). The dot size represents the number of overlapped kinase motifs and dot color shows enrichment factors. One-way ANOVA with Tukey’s test (**e**, **f**); two-tailed unpaired *t*-test (**k**, **l**); two-sided Chi-square (**n**). *P < 0.05; †*P* < 0.01; ‡*P* < 0.001.

Several hours of acute sleep deprivation abolish the daily phosphorylation cycle and induce time-dependent cumulative phosphorylation of the brain/synaptic proteome^24,25^. To characterize whether the phosphorylation also accumulates in a Pr-SD time-dependent manner or is associated with the PONE status — and to identify potential molecular changes corresponding to the decline in brain state and the PONE status — we performed quantitative phosphoproteomic and proteomic studies using multiplex tandem mass tag (TMT) labeling on an Orbitrap platform^24,26,27^. Brains from AD, PS and PD groups were collected under the SWAM model at parallel time points (Fig. 3a and Supplementary Tables S2, S3). Phosphoproteomic analyses revealed substantial alterations at the phosphopeptide level (Q < 0.2 and |log_2_(change)| > 0.3) in both the PD/PS comparison (5.1% of 29,436) and the PD/AD comparison (19.9% of 29,373) (Fig. 3b–d). Intriguingly, such changes were absent in the PS/AD comparison, even though PS and PD mice underwent the same SWAM duration (Fig. 3b). This indicates that brain phosphoproteomic changes were independent of SWAM duration or corresponding stress effects, but rather associated with PONE status.

In contrast to the changes observed between 6-h complete deprivation of sleep (SD6) and 6-h ad libitum sleep (S6)^24^, the faint shifts in PS/AD comparison could be attributed to the intermittent sleep bouts during Pr-SD (Fig. 1e and Supplementary Discussion 3), which led to a natural restoration of sleep need in the Pr-SD condition and accompanying phosphoproteomic alterations^28^. However, phosphopeptides that showed notable changes in the PD/AD comparison also displayed significant group differences in the PS samples (Fig. 3e, f). This suggests a delicate equilibrium of brain phosphoproteins becoming unsettled during the PS state and transforming into significant disruptions following the onset of PONE status in the PD state. Proteomic analyses showed only a few quantified proteins exhibited significant changes in PS/AD (0.95% of 9069), PD/PS (0.31% of 9075) and PD/AD (2.2% of 9060) comparisons, which were different from the phosphoproteomic changes in terms of degree and pattern, and made minimal contributions to phosphopeptide changes (Supplementary Fig. S3a–i and Table S3). Besides, gene ontology (GO) analysis also revealed that the proteomic-enriched components, such as fibrinogen complex and lipoprotein particle (Supplementary Fig. S3j, k and Table S4), were completely different from phosphoproteomic analyses (Supplementary Fig. S4a).

Our previous study found both SD6 and *Sleepy* (*Slp*) mutant mice exhibit a global hyperphosphorylation in the comparisons of brain proteomes between SD6 and RS3 (SD6 followed by a 3-h recovery sleep) or S6, Slp and wild-type (WT) samples^24^. We have identified a core set of phosphopeptides and hyper-phosphoproteins as the shared substrates in the two increased sleep need models (SD6 and *Slp*) that function as the molecular substrates of sleep need^24,29^. By applying unsupervised cluster analysis to 317 shared peptides in all three models (SD6, *Slp*, and Pr-SD), PD samples were grouped with high sleep need group (SD6 and Slp), indicating the response of sleep need-related phosphopeptides to Pr-SD (Fig. 3g, h). These 317 peptides could be further classified into 3 classes (A–C) based on patterns of abundance changes (Fig. 3h). Subsequently, we conducted phosphorylation state change (ΔPs) analysis to elucidate functional alterations of phosphoproteins with multiple phosphorylation sites^24,30^ (Supplementary Table S5). 165 Hyper-proteins (ΔPs > 2.4) were observed in Pr-SD model, with 43 in the PD/PS comparison and 156 in the PD/AD comparison, as well as a profound number of Hypo-proteins (ΔPs < 2.4) (Fig. 3i, j). In line with the clustering results, the sleep-need-index phosphoproteins (SNIPPs), whose phosphorylation states closely mirror the shifts in sleep need^24^, demonstrated notably elevated ΔPs values in both PD/PS and PD/AD comparisons (Fig. 3k, l and Supplementary Discussion 4). We termed the 47 hyper-proteins shared in all 3 sleep models as PD-SNIPPs, to highlight the SNIPPs that corresponded to PONE status (Fig. 3m). Moreover, functional analysis revealed most of the class A–C phosphopeptide-corresponding proteins, and Hyper-proteins were associated with synaptic functions, as indicated by our curated synaptic protein database (Fig. 3n and Supplementary Table S6). GO analysis showed that these proteins were enriched in similar synaptic categories (Supplementary Fig. S4a and Supplementary Table S4). All this evidence indicates a wide range of synapse- and sleep-related proteins were impacted during PONE status, and the KEGG pathway enrichment analysis conducted on the 192 core molecular interface proteins (class A–C and PD-SNIPPs) also supported this finding (Supplementary Fig. S4b and Table S4). Specifically, pathways such as glutamatergic, endocytosis, synaptic vesicle release, long-term potentiation, cAMP and calcium signaling were particularly relevant. Several core genes — such as metabotropic glutamate receptor 5 (*Grm5*)^31,32^, SH3 and multiple ankyrin repeat domains protein 3 (*Shank3*)^33^, and voltage-dependent R-type calcium channel subunit alpha-1E (*Cacna1e*)^34^ — previously identified as sleep regulators and associated with synaptic plasticity, were also highlighted.

In addition to the analysis of phosphoprotein levels, we further employed a newly developed method for motif analysis to investigate the relevant changes of kinase signaling pathways^35^. A tremendous kinase disturbance in PD samples was observed, in which 174 of 303 kinase motifs were significantly enriched (127) or depleted (47) (Supplementary Fig. S4c and Table S7). To comprehensively explore the kinase signaling patterns in PD samples, we curated 20 phosphoproteomic datasets with diverse profiles, encompassing scenarios such as pharmacological^36–40^ or genetic neuron manipulation^41^, behavior studies^42,43^, and cellular damage^44^ (Supplementary Fig. S4d). All datasets were reprocessed using an identical standard and then subjected to kinase motif analysis, minimizing potential discrepancies to the greatest extent (Supplementary Table S7). Next, we conducted a Functional Quadrant Enrichment Analysis (FQEA) to evaluate the kinase signaling similarity across all 20 datasets. In brief, kinases showing a significant change in both datasets were split into 2 diagonal quadrant groups based on their log_2_-transformed frequency factors (Supplementary Fig. S4c, e and f). For example, kinases in the 1^st^ and 3^rd^ quadrants were classified into a group with a positive correlation (Supplementary Fig. S4f). Subsequently, chi-square analysis was performed with the number of kinases in a specific group, against the background distribution of kinase points across all datasets (Supplementary Fig. S4f).

FQEA analysis effectively evaluated the similarity of kinase disturbance, evident from the strong negative correlation between tetrodotoxin (TTX) and bicuculline (BIC) datasets, aligning with their respective roles in synaptic up- or down-scaling process^36,37^ (Supplementary Fig. S4e, g and h). In the case of PD datasets (PD/AD and PD/PS), a substantial number of kinase motifs displayed positive correlations with sleep-related and BIC- or Ca^2+^-treated datasets (Supplementary Fig. S4i), suggesting the relevant kinase signaling pathways were altered in the brain of PD mouse. Conversely, the lack or negative pattern of significant correlations between PD and other datasets revealed distinct kinase signaling pathway patterns, particularly in comparison with stress-related models (chronic unpredictable mild stress, CUMS and forced swim stress, FSS) and DNA damage (IR-A549) (Supplementary Fig. S4i). Importantly, it is worth noting that the correlation among datasets did not depend on the total number of overlapped kinases, but rather on the distribution of kinase points within distinct diagonal quadrant groups (e.g., in the first line, the number of overlapped kinases of TTX-R-24h was more than SRPK2-OE obviously, but no significant correlation was observed) (Supplementary Fig. S4i).

BIC induces homeostatic synaptic downscaling by blocking inhibitory synaptic transmission^45^. The highly positive correlation in kinase signaling pathways between PD and BIC samples demonstrated the significant downscaling of synapses in the Pr-SD brain (Supplementary Fig. S4f, i). This suggested chronically elevated network activity and reduced neuronal firing rates in PD brains, which were in line with our electrophysiology results (Fig. 2c–e). Besides, we analyzed the phosphoproteomic changes of potassium, calcium, sodium, manganese ion and proton transporters (Supplementary Table S6). 54% (116 out of 213) ion channels showed significant phosphorylation changes (Supplementary Fig. S5a–f). Collectively, these findings reveal a profound disturbance of phosphoproteome and kinase signaling pathways in PONE status, which are closely associated with sleep regulation and neuronal activity.

The contribution of different brain areas to sleep regulation varies, raising the question of whether PONE-related phosphoproteomic disturbances differ across brain regions. While multiple studies on the forebrain have provided valuable insights into sleep regulation, knowledge of the cerebellum remains limited. To address this gap, we conducted phosphoproteomic analyses of cerebellum samples from AD, PS, and PD groups (ADc, PSc, and PDc) (Supplementary Fig. S6a and Table S8). Given the smaller scale of the cerebellum experiment relative to the whole brain, we applied the corresponding *P* value cutoff of PD/AD comparison to the cerebellar analyses (Fig. 3d and Supplementary Fig. S6b). In the PDc/PSc and PDc/ADc comparisons, 6.6% and 10.3% of phosphopeptides, respectively, exhibited significant changes (Supplementary Fig. S6c, d). Notably, phosphopeptide changes in the cerebellum strongly correlated with those in the whole brain in the PD/AD comparison (Supplementary Fig. S6e), indicating comparable changes in phosphopeptide levels between the two regions. ΔPs analysis further supported this, revealing 72 hyper-phosphorylated and 86 hypo-phosphorylated proteins in the PDc/ADc group (Supplementary Fig. S6f, g). Additionally, SNIPPs exhibited elevated ΔPs values in both the PDc/PSc and PDc/ADc groups, suggesting that sleep-need–related phosphorylation dynamics also occur in the cerebellum (Supplementary Fig. S6h).

Next, we compared the extent of phosphoproteomic changes in PD mice to other datasets. Overall, the PD samples exhibited the most drastic changes in both phosphopeptide and phosphoprotein levels (Supplementary Fig. S6i). This indicates that the impact of Pr-SD and PONE status on the phosphoproteome was even greater than the effects of BIC treatment on cultured neurons. Notably, BIC treatment for 24 h could potentially be lethal to the animals, implying that the phosphoproteomic alterations observed in PD mice may reflect a more profound and widespread disruption than what could be induced by BIC treatment in vivo. This suggests that the phosphorylation-based balance across all brain regions may be significantly disrupted in PD mice. Therefore, we performed kinase enrichment analyses on cerebellum samples, revealing significant enrichment or depletion in nearly 50% of kinases (Supplementary Fig. S6j, k). FQEA analysis further confirmed a similar pattern of kinase pathway changes between cerebellum samples and whole brain or BIC-treated samples (Supplementary Fig. S6l–o). In the SD6 and *Slp* models, we identified 53 core sleep-regulating kinases that were significantly altered across all three comparisons (Supplementary Fig. S6p). Many of these kinases were also found in the PD/AD and PDc/ADc comparisons, including kinases from the AMP-activated protein kinase (AMPK) family, with-no-lysine [K] kinases (WNKs), and cGMP-dependent protein kinases (PRKGs), all of which have been implicated in sleep regulation (Supplementary Fig. S6p). These findings suggest that the cerebellum of PD mice also undergoes significant phosphoproteomic disruption, underscoring the widespread impact of Pr-SD on the brain.

### SLP and PP2A affect both sleep and PONE status development

**Fig. 4:**
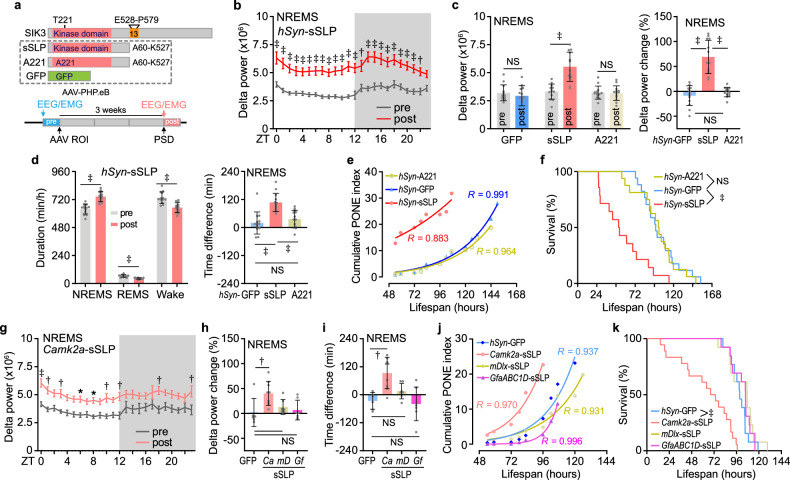
The effect of SLP on sleep and PONE status development. **a** Schematic diagram of the protein structure of SIK3 and truncated proteins for AAV expression, and the experimental design for EEG/EMG recording and survival tests. **b**–**d** Sleep analyses for *hSyn-GFP*, *hSyn-A221* and *hSyn-sSLP* group (*n* = 13 per group). 24-h absolute delta power of NREMS before (pre) and after (post) *hSyn-sSLP* injection (**b**). Mean absolute NREMS delta power (left) and the change ratio [(‘post’- ‘pre’)/ ‘pre’] (right) in mice injected with *hSyn-GFP*, *hSyn-A221* and *hSyn-sSLP* (**c**). Duration (left) and time difference (right) of NREMS in mice injected with *hSyn-GFP*, *hSyn-A221* and *hSyn-sSLP* (**d**). **e**, **f** Analysis of cumulative mean PONE index (**e**) and survival (**f**) of SWAM mice injected with *hSyn-GFP* (*n* = 17), *hSyn-A221* (*n* = 16) and *hSyn-sSLP* (*n* = 14). **g***–***i** Sleep analyses for *hSyn-GFP* (*n* = 6), *Camk2a-sSLP* (*n* = 9), *mDlx-sSLP* (*n* = 7) and *GfaABC1D-sSLP* (*n* = 7). 24-h absolute delta power (**g**); change ratio of mean absolute NREMS delta power (**h**); time difference of NREMS duration (**i**). **j**, **k** Analysis of cumulative mean PONE index (**j**) and survival (**k**) of SWAM mice injected with *hSyn-GFP* (*n* = 13), *Camk2a-sSLP* (*n* = 18), *mDlx-sSLP* (*n* = 13) and *GfaABC1D-sSLP* (*n* = 13). Data are mean ± s.e.m. Two-way ANOVA with Sidak’s test (**b**, **g**); two-tailed paired *t*-test (**c**, **d,** left); one-way ANOVA with Tukey’s test (**c**, **d,** right); log-rank (Mantel-Cox) test (**f**, **k**); one-way ANOVA with Dunnett’s test (**h**, **i**). *P < 0.05; †*P* < 0.01; ‡*P* < 0.001; NS, not significant, *P* > 0.05. *n* refers to the number of biological replicates.

The kinome and phosphatome in PD samples both exhibited profound alterations (Supplementary Fig. S7a, b and Table S6), which, together with the significant disturbance in kinase signaling under PONE status, led us to question whether the surge in brain phosphorylation, resulting from the dysfunctions of kinase and phosphatase, could affect PONE status and sleep regulation. Fortunately, *Slp* mutant mice (lacking exon 13 of *Salt-inducible kinase 3*, *Sik3*) exhibit relevant phosphoproteomic changes and sleep phenotypes^24,29^, providing a valuable approach to experimentally induce phosphoproteomic disturbances in the mouse brain. Hence, we delivered a truncated form of SLP (sSLP, A60-K527, the catalytic domain of SLP) into the mouse brain using the AAV-PHP.eB virus expression system, driven by the human *Synapsin* (*hSyn*) gene promoter^46^ (Fig. 4a and Supplementary Discussion 5). sSLP expression closely recapitulated the excessive sleep need observed in *Slp* mutant mice, characterized by elevated NREMS delta power and duration^29^, which were absent in the case of A221, a kinase activity-deficient variant of sSLP (Fig. 4a–d and Supplementary Fig. S7c, d). During the SWAM test, sSLP expression led to notably quicker PONE status development and shorter survival duration compared to GFP and A221, demonstrating the necessity of kinase activity for this phenotype (Fig. 4e, f and Supplementary Fig. S7e). Furthermore, we expressed active sSLP under the control of *Camk2a*, *mDlx*, or *GfaABC1D* promoters to target excitatory neurons, inhibitory neurons, and astrocytes, respectively. Among these, only *Camk2a* promoter-driven sSLP expression resulted in an increase in sleep need and a reduction in the duration of SWAM (Fig. 4g–k and Supplementary Fig. S7f–h). Collectively, we have showcased that the heightened activity of kinase in excitatory neurons promotes the development of PONE status in the SWAM model.

**Fig. 5:**
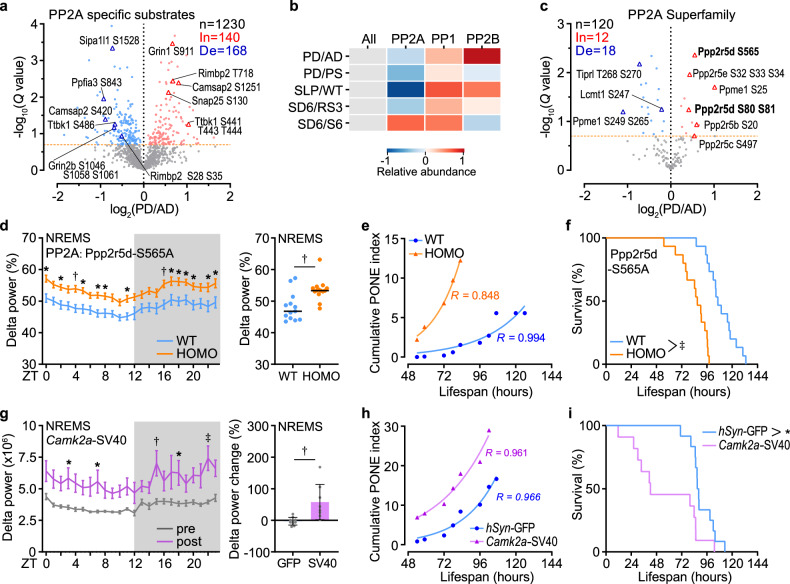
The effect of PP2A on sleep and PONE status development. **a** Volcano plots of quantified phosphopeptides of PP2A specific substrates in PD/AD comparison. **b** Relative abundances of the specific substrates for three phosphatases in sleep-related comparisons. **c** Volcano plots of quantified phosphopeptides of PP2A family in PD/AD comparison. **d** 24-h (left) and mean (right) relative NREMS delta power of *Ppp2r5d* (S565A) homozygous (HOMO, *n* = 14) and WT (*n* = 13) littermates. **e**, **f** Analysis of cumulative mean PONE index (**e**) and survival (**f**) for *Ppp2r5d* (S565A) littermates (*n* = 15 per group). **g** 24-h absolute delta power (left) and the change ratio (right) of NREMS in mice injected with *hSyn-GFP* (*n* = 10) and *Camk2a-SV40* small T antigen (*n* = 8). **h**, **i** Analysis of cumulative mean PONE index (**h**) and survival (**i**) of SWAM mice injected with *hSyn-GFP* (*n* = 12) and *Camk2a-SV40* (*n* = 11). Data are mean ± s.e.m. Two-way ANOVA with Sidak’s test (**d**, **g,** left); two-tailed unpaired *t*-test (**d**, **g,** right); log-rank (Mantel-Cox) test (**f**, **i**). *P < 0.05; †*P* < 0.01; ‡*P* < 0.001; NS, not significant, *P* > 0.05. *n* refers to the number of biological replicates.

In addition to kinase dynamics, phosphatases are another crucial factor in maintaining the balance of phosphorylation in the brain. Given the substantial number of dephosphorylated peptides in PD samples (Fig. 3c, d), our hypothesis posited that the disruption of PONE status-related phosphoproteomic alterations might result from excessive phosphatase function. To systematically identify potential fluctuation in phosphatase function, we first conducted a comprehensive position-specific scoring matrix (PSSM) search across the entire proteome. This involved utilizing three well-established short linear motifs (SLiMs) associated with phosphatases (LxxIxE motif for PP2A, RVxF motif for PP1, and LxVP and PxIxIT motifs for PP2B) to explore their likely substrates^47,48^ (Supplementary Fig. S8a and Table S9). Then, by computing the relative abundance of phosphatase-specific substrates, we observed that only PP2A consistently showed signs of elevated activity, as indicated by the relatively reduced abundance of its substrates (Fig. 5a, b). Moreover, we also found increased phosphorylation levels at Ser80/81 and Ser565 of PPP2R5D, a regulatory subunit of PP2A, which has been associated with enhanced phosphatase activity in previous studies^49,50^ (Fig. 5c). Therefore, to confirm the effect of phosphatase dysfunction on sleep regulation and the development of PONE status, we introduced a single amino acid substitution in *Ppp2r5d* (S565A) using the CRISPR/Cas9 genome editing technology (Supplementary Fig. S8b). S565A mutant mice exhibited constitutively increased NREMS delta power, while maintaining normal sleep duration (Fig. 5d and Supplementary Fig. S8c, d), indicating a unique role of PPP2R5D in sleep regulation. Echoing the EEG findings, S565A mutant mice showed accelerated PONE status development and shortened lifespan in SWAM test (Fig. 5e, f and Supplementary Fig. S8e). Furthermore, the effects of PP2A on sleep and PONE status were operative in excitatory neurons, as illustrated by inhibiting PP2A activity with *Camk2a* promoter driven SV40 small T antigen AAV system^51^ (Fig. 5g–i and Supplementary Fig. S8f–i). By contrast, mice with point mutations in *Ppp1r14a* S26A (a regulatory subunit of PP1), *Ppp3ca* S462A or *Ppp3cb* S472A/S479A (2 catalytic isoforms of PP2B) exhibited neither sleep abnormalities nor PONE status development aberration (Supplementary Fig. S8j-q), despite the phosphatase activity of PP1 declined concordantly across all sleep models (Fig. 5b). S565A mice exhibited subtle changes in brain phosphoproteomics, with 85% of peptides showing increased phosphorylation levels (Supplementary Fig. S9a, b and Table S8). Moreover, 14 proteins, including SNIPPs such as Protein piccolo (PCLO) and Microtubule-associated protein 2 (MAP2), were identified as hyper-phosphorylated (Supplementary Fig. S9c, d). These findings align closely with the elevated NREMS delta power observed in S565A mutant mice. Additionally, an increase in phosphorylation levels was noted in PP2A substrates (Supplementary Fig. S9e), reflecting altered phosphatase activity of PP2A. KEGG analysis of proteins with significantly altered peptides revealed that pathways such as the cAMP signaling pathway and dopaminergic synapse were impacted by the S565A mutation in PPP2R5D (Supplementary Fig. S9f). Based on these results, we identified the dual roles of SLP and PP2A in sleep regulation and PONE status development, serving as prominent instances within the wide spectrum of kinase-phosphatase dysregulations observed in PD samples.

### Brain phosphorylation balance reconstruction resists PONE status development

**Fig. 6:**
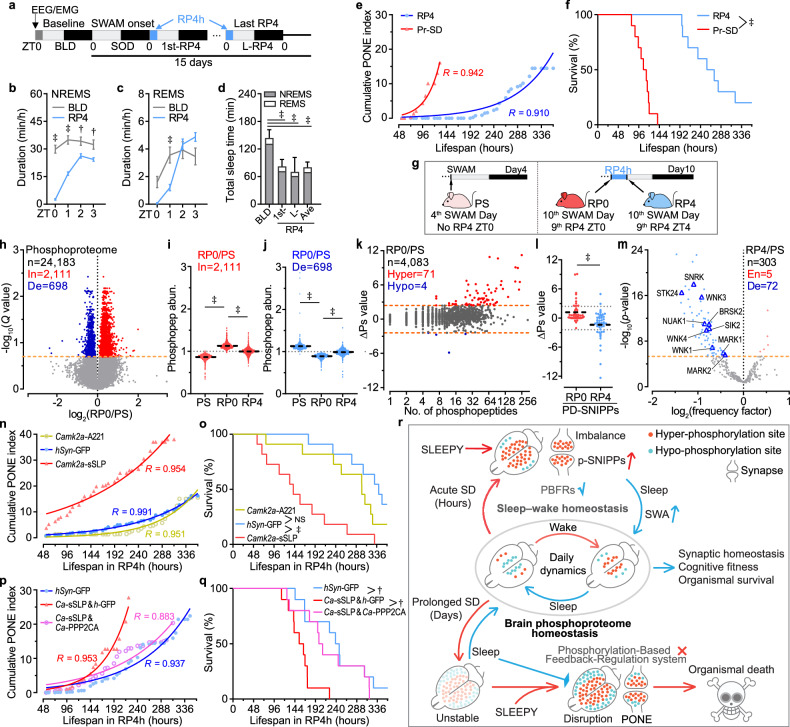
The effect of brain phosphoproteomic reconstruction on PONE status development. **a** Schematic of the 4-h recovery period (RP4h) experiment. **b**, **c** Analysis of hourly duration of NREMS (**b**) or REMS (**c**) for BLD (ZT0-3) and the average of all RP4 (*n* = 16). **d** Total sleep time on BLD (ZT0–3), 1st-RP4, L-RP4 (last-RP4) and the average (Ave) of all RP4 (*n* = 16). **e**, **f** Cumulative PONE index (**e**) and population survival (**f**) of SWAM mice without (SWAM) or with recovery (RP4, *n* = 10 per group). **g** Schematic diagram of the RP4h phosphoproteomic study. **h** Volcano plot showing all phosphorylation changes in the RP0/PS group. In, increased; De, decreased. **i**, **j** Mean abundance analysis of increased (**i**) and decreased (**j**) phosphopeptides in the RP0/PS comparison under three conditions. **k** Global ΔPs analysis in RP0/PS comparison. **l** Comparative ΔPs analysis of 47 PD-SNIPPs between RP0/PS and RP4/PS comparisons. **m** Global kinase motif enrichment analysis of phosphoproteomic data from RP4/PS groups. En, enriched; De, depleted. **n**–**q** Cumulative PONE index (**n**, **p**) and survival analyses (**o**, **q**) of SWAM mice with RP4h and injected with AAV. *n* = 11 (**n**, **o**, GFP, sSLP and A221); *n* = 10 (**p**, **q** GFP and GFP&sSLP); *n* = 9 (**p**, **q** GFP&PPP2CA). **r** A model illustrating homeostasis of brain phosphoproteome linking sleep regulation and functions. Data are mean ± s.d. Two-way ANOVA with Sidak’s test (**b**, **c**); one-way ANOVA with Tukey’s test (**i**, **j**); log-rank (Mantel-Cox) test (**o**, **q**); two-tailed unpaired *t*-test (**l**). *P < 0.05; †*P* < 0.01; ‡*P* < 0.001; NS, not significant, *P* > 0.05. *n* refers to the number of biological replicates.

Recovery sleep immediately following SD6 is accompanied by global dephosphorylation of brain proteome^24^, which may function as an essential time window to restore the overall molecular and neuronal networks challenged by previous waking experiences and maintain the organismal fitness. To further characterize the relationship between sleep, phosphoproteomic disturbance, and PONE status, we introduced a 4-h recovery period (RP4h) to SWAM-exposed mice since the 2^nd^ SWAM day during a fixed time zone (ZT0 to ZT4) (Fig. 6a). EEG/EMG analysis showed that within the RP4h, there was ~80 min of recovery sleep (NREMS 69.7 ± 9.4 min; REMS 10.5 ± 2.8 min), which was much shorter compared to baseline sleep (Fig. 6b–d, Supplementary Fig. S10a, b and Discussion 4). We found a significantly delayed onset of PONE status and mortality under the condition of RP4h, with several mice remaining alive even after 15 days (Fig. 6e, f). Introducing classical sleep deprivation procedure during RP4h completely abolished the delay in mortality, which strongly demonstrates the primary role of sleep in the curative effect of the recovery period (Supplementary Fig. S10c).

To examine whether this minimal amount of recovery sleep could also restore the phosphorylation state of brain proteome, we harvested mice brains in three conditions and all mice were evaluated as being in the PS state: 1) the 4th SWAM day without RP at ZT0 (PS); 2) the 10th SWAM day at ZT0, the beginning of the 9th RP (RP0); 3) the 10th SWAM day at ZT4, the end of the 9th RP (RP4) (Fig. 6g). We observed 2,111 hyper-phosphorylated peptides (*Q* < 0.2) in RP0 samples, whereas alterations in RP4 were the least pronounced (Fig. 6h and Supplementary Fig. S10d). A significant restoration of phosphopeptides abundance was detected in RP4, including both increased and decreased ones of RP0/PS comparison (Fig. 6i, j). This subtle improvement effectively preserved a phosphoproteomic profile resembling that of the PS state, which was considered a causal factor in the delayed development of PONE status (Fig. 6e, f). The phosphoproteomic fluctuations between RP0 and RP4 showed patterns similar to the phosphorylation changes observed in SD6 models, where brain phosphorylation levels varied dynamically in response to sleep need^24^. This indicates a fragile sleep homeostasis (or brain phosphorylation balance) develops in response to the recovery sleep (Fig. 6i, j). ΔPs analysis further confirmed the restorative effect at the phosphoprotein level, particularly highlighted by the declined ΔPs value of PD-SNIPPs (Fig. 6k, l and Supplementary Fig. S10e–i). The hyper-proteins of RP0/PS and hypo-proteins of RP4/PS were highly enriched in synaptic-related GO items (Supplementary Fig. S10j–l). Besides, we also identified a set of functionally depleted kinases in RP4 samples, in contrast to their functional enrichment observed in PD samples (Fig. 6m and Supplementary Fig. S4c). These combined findings on phosphopeptides, phosphoproteins and kinase signaling pathways collectively confirm the restorative effect of the recovery sleep on the brain phosphoproteome in Pr-SD-treated mice.

Next, we examined the effect of RP4h on sSLP expression mice to figure out the curative impact of recovery sleep on kinase dysfunction. The results showed that RP4h extended the SWAM duration of sSLP expression mice, and delayed PONE status development (Fig. 6n, o). However, the SWAM defect remained evident in sSLP mice, compared to A221 and GFP (Fig. 6n, o). This partial rescue was probably due to the exorbitant kinase activity of sSLP, which exceeded the modulation capacity of RP4h. Therefore, in order to counteract excessive kinase activity, we co-expressed a series of PP2A phosphatase activity agonists, including PPP2R5D, leucine carboxyl methyltransferase 1 (LCMT1)^52^ and the catalytic subunit of PP2A (PPP2CA) in sSLP expression mice. All these PP2A agonists prolonged the survival duration to some extent in RP4h treatment, with PPP2CA, in particular, fully rescuing mice from accelerated mortality and normalizing the development of PONE status (Fig. 6p, q and Supplementary Fig. S10m, n). Notably, immunoblotting with antibodies against 3 phosphorylated substrate motifs showed that, complementary expression of PPP2CA significantly restored global phosphorylation of substrates of AMPK, protein kinase C (PKC) and “ataxia telangiectasia mutated” (ATM) compared to sSLP expression (Supplementary Fig. S10o). In addition, we selected five antibodies targeting specific phosphorylation sites to confirm the changes in the brain following sSLP and PPP2CA overexpression, including HDAC4, a substrate of SIK3 (Supplementary Fig. S10p). The results showed that phosphorylation levels of all five sites increased after sSLP expression and significantly decreased in the PPP2CA co-expression samples (Supplementary Fig. S10p). Taken together, these findings demonstrate that sleep facilitates reconstruction of the brain phosphoproteome following Pr-SD, highlighting the pivotal role of kinase-phosphatase balance in the development of PONE status.

## Discussion

It has been proposed that the core sleep functions should be tightly linked to the homeostatic regulation of sleep through an undefined molecular interface^9–11^. Together with recent phosphoproteomic studies on sleep^24,25,32,53,54^, evidence from varying experimental paradigms and multiple temporal dimensions indicates that the brain/synaptic phosphoproteome is homeostatically regulated across sleep–wake cycles, and that disturbance caused by forced awakening must be restored by the following recovery sleep. Functional analyses further suggest that the core sleep functions, such as maintaining synaptic homeostasis and cognitive fitness during daily circumstances, and safeguarding survival in the face of long-lasting sleep loss are mechanistically linked to the restorative role of sleep on phosphoproteome homeostasis, which also underlies the regulation of sleep–wake homeostasis^24,25,32,53,54^. Thus, we posit that an intrinsic phosphorylation-based feedback regulation (PBFR) system^9,53^ is hijacked by both homeostatic regulation of sleep and the core effects induced by sleep loss, including sleep need^24,25,53^, synaptic functions^54^, and organismal survival (Fig. 6r). The regulators and effectors of the PBFR system may represent a parsimonious molecular interface linking sleep regulation and such fundamental cellular/systemic functions.

The decrease in NREMS delta power during SWAM was a counterintuitive result, contrary to expectations based on research into classical sleep deprivation, where an increase was anticipated (Supplementary Discussion 4). We interpreted this as an indication that the quality of sleep was poor due to frequent awakenings. Besides, previous studies have reported a blunted increase in NREMS delta power when sleep deprivation exceeds 9 h^28^. Accordingly, we reasonably hypothesized that there might be a natural restoration/resilience mechanism counteracting the elevation of NREMS delta power, which could also be operative in the current case. Moreover, short sleep occurred but was severely disrupted or inhibited during SWAM, potentially leading to a suppression or disruption of NREMS delta power and sleep homeostasis. This, in turn, may have resulted in the loss of sleep ability at the PONE status. Notably, the biological role of NREMS delta power remains largely unknown, and the question of whether it is merely an epiphenomenon or serves some mysterious functions remains unanswered despite years of research. Our results may offer new insights and raise awareness regarding the significance of NREMS delta power and the sleep homeostasis it may represent.

## Materials and methods

### Animals

All animal experiments were performed according to procedures approved by the Institutional Animal Care and Use Committee of Harbin Institute of Technology (HIT/IACUC). Unless otherwise stated, all mice were 8–12 weeks males in C57BL/6 J background and housed under humidity (50 ± 10%), light (12-h light/dark cycle) and temperature (24 ± 2 °C) controlled conditions with free access to water and food. Point mutation mice (*Ppp2r5d* S565A, *Ppp1r14a* S26A, *Ppp3ca* S462A and *Ppp3cb* S472A/S479A in C57BL/6 L background) were designed and generated at Cyagen Bioscience (Guangzhou, China).

### Pr-SD

Pr-SD was conducted based on rodents’ (rats or mice) aversion to water environments, as they typically avoid sleeping in water^22,23^. To achieve continuous sleep deprivation, we employed the SWAM. In this method, mice were housed individually in rectangular cages filled with ~0.6 cm of sterile water. During the SWAM onset day, mice entered a novel environment basically and explored the cage at the beginning of several hours. After that, the thin layer of water prevented mice from entering into deep sleep and woke mice when their noses touched the water, which caused a significant sleep duration reduction.

To ensure animal welfare, several measures were taken. All mice were housed in clean and spacious cages prior to the experiments, with a two-day acclimation period to allow them to adjust to single housing. During the experiments, cages and water were replaced every 24 h following the PONE index rating. Additionally, the mice were monitored continuously for survival via camera. Survival time was defined as the total hours from the start of sleep deprivation until spontaneous organismal death (immobility for over 5 min) or the time point of euthanasia.

In line with animal welfare guidelines, mice that reached PONE status after index rating were euthanized at three specific time points each day to alleviate their suffering, except for experiments directly related to establishing the PONE index system.

### PONE status evaluation

Variables used for subjects’ status evaluation were adopted from disk-over-water studies^17–19,23^. Four behavioral variables were rated on a 3-point scale (0, 1 and 2) represent the increased severity of debility. Value 0 or 2 was assigned when subjects were in a “pretty healthy” or “extremely feeble” state, while value 1 was assigned when subjects were in an “intermediate” state, encompassing all ambiguous conditions that were less confidently categorized as any of the two boundary states. The “pretty healthy” and “extremely feeble” states of each variables were defined as following. (1) frequency and duration for forepaws underwater: less than 2 s in an observation of 5 s; more than 10 s in an observation. (2) status of mouth stick with water: totally dry; stuck with an obvious droplet. (3) response after gentle touch: run away rapidly in 2 s; not run away in 10 s. (4) food consumption ratio compared with the same time zone of previous day: > 60%; < 30%. Due to mice were frequently climbing onto the food rack of cage lid on the 1st SWAM day, new chow diet was replaced and weighted at ZT 0 of the 2nd SWAM day, thus the first full PONE index can be calculated at ZT6 of the 3rd SWAM day. When previous day’s food consumption value was not available, the average food consumption during the same period of the 2nd SWAM day of WT mice was used for calculation. Rating was performed at fixed time point, ZT0, ZT6 and ZT11 for each animal, and the PONE index value was calculated as the sum of all rating values at indicated ZT.

When baseline variables were measured, such as body weight and basal food intake, mice were individually housed at least 3 days for adaptation, and 1-day baseline measurements before SWAM were used as basal condition. When analysis the cumulative effect of PONE value, the mean PONE value for each experimental group was calculated and used for exponential growth regression analysis. When analysis the time-dependent development of PONE index, the last four PONE index values for each subject were aligned within a relative 24-h timeline and analyzed by segmental linear regression to identified the inflection point. The remaining survival duration was calculated from each rating time to the actual death time, when two sequential ratings shown same PONE index value or later one smaller, only the duration from the last one was used. For the 4-h recovery period (RP4h) experiments, mice were individually housed at least 3 days and resided in their homecages during recovery. The recovery was performed since the 2nd SWAM day at ZT0 for 4 h per day and released all survived subjects after the 15th SWAM day. Representative results and analyses of PONE index were listed in Supplementary Table S1.

### EEG recording and sleep phenotype analysis

The sleep phenotype analysis was performed as previously described^24,29,55^. Under anesthesia by isoflurane (3% for induction and 1% for maintenance), 8–10 weeks old mice were implanted with EEG/EMG electrodes under stereotaxic control. After surgery, mice were individually housed and allowed a minimum 1-week recovery. Mice were then acclimatized to the recording chamber around 1-week before EEG/EMG signal recording. For SWAM and RP4h experiments, 3 days baseline EEG/EMG recording were conducted and the day before SWAM onset was used as basal condition. EEG/EMG signals were monitored continuously. Cleaned cage and water was replaced every 24 h. Mice were returned to their homecages during recovery. For AAV experiments, baseline EEG/EMG recording was conducted on WT mice, then randomly divided into different groups and injected with according AAVs as indicated. Mice remained in the same recording chambers for 3 weeks, then EEG/EMG signals were recorded.

Raw EEG/EMG recording data were subjected to semi-automated analysis followed by manual correction. Fast Fourier transform analysis was used to decompose EEG signals to 1 Hz bins from 1 to 30 Hz for following analysis. 20 s epoch was used to characterize wake (low amplitude and fast EEG, high amplitude and variable EMG), REMS (dominant 5–8 Hz theta band EEG and EMG atonia), or NREMS (high amplitude 1–4 Hz delta EEG and low EMG tonus) state. Absolute (arbitrary units) EEG power density was determined by the delta (1–4 Hz), theta (5–8 Hz), alpha (9–14 Hz), and beta (15–30 Hz) band activities during wake, REMS, or NREMS state for indicated period. In circadian variation plots, each data point represents the mean value of either duration or EEG power density in the following 1 hour for the corresponding wake, REMS, and NREMS state. Researchers were blinded to the treatments during data analysis and only animals with unreadable EEG signals were excluded.

The LFD was defined as the last complete ZT0-23 before the death event. The L-24h was defined as a complete 24 h before the death time point. For, example, if a mouse dies at ZT2 of Day 4, “LFD” refers to Day 3, representing the last complete time zone from ZT0 to ZT23. “L-24h” refers to the 24-h period leading up to death, from ZT2 of Day 3 to ZT2 of Day 4.

### Mass spectrometry sample preparation

Mass spectrometry samples were prepared as previously described with modifications^24^. Mouse tissues were quickly dissected, flash frozen in liquid nitrogen and stored in –80 °C. For the Pr-SD model, whenever PD mouse was identified, whole brain from PS and AD mice were collected to ensure PS and PD subjects experienced the same SWAM duration. For the RP4h model, all mice used were evaluated as in PS state and brains were harvested at three conditions: 1) the 4th SWAM day at ZT0 without recovery period (PS); 2) the 10th SWAM day at ZT0, the beginning of the 9th recovery period (RP0); 3) the 10th SWAM day ZT4, the end of the 9th recovery period (RP4).

Typically, 5 mL of lysis buffer (50 mM HEPES (pH 7.4), 150 mM NaCl, 2 mM MgCl_2_, 1% SDS) was used for homogenization of one whole brain in a glass tissue homogenizer. Protein lysates was mixed with 50 U/mL of Universal Nuclease (Thermo Scientific), and then added 2 mL 10% SDS buffer. Protein homogenates were incubated for 30 min and centrifuged at 15,000 *g* for 20 min at room temperature, supernatant was transferred to a new 15 mL tube. Bicinchoninic acid (BCA) assay was used to determine protein concentration (Thermo Scientific). Protein lysates were reduced with dithiothreitol and alkylated with iodoacetamide, precipitated by chloroform–methanol precipitation method and then resuspended in 8 M urea buffer. Protein lysates were digested with lysyl-endopeptidase (Lys-c) (Wako Pure Chemical Industries) for 2 h, followed by dilution to 2 M urea with 25 mM ammonium carbonate buffer (pH 7.8), and trypsin (Promega) digestion overnight at room temperature, 1% trifluoroacetic acid (TFA) used to stop the digestion. Peptide mixtures were subjected to C18 solid-phase extraction (Sep-Pak, Waters) and then vacuum-centrifuged to near-dryness.

Desalted peptides from 8 mg protein lysates for each sample were resuspended in 1 mL phosphopeptide binding buffer (2 M lactic acid/50% acetonitrile (ACN)) and centrifuged at 15,000 *g* for 20 min; supernatant was transferred to a new tube for phosphopeptides enrichment. Titanium dioxide (TiO_2_) beads (GL Sciences) were washed for three times with phosphopeptide binding buffer. Equal amount TiO_2_ beads were added to each peptide mixtures and incubated with gentle rotation for at least 1 h. Then TiO_2_ beads were washed twice with phosphopeptide binding buffer, twice with wash buffer (50% ACN/0.1% TFA). Finally, phosphopeptides were eluted twice with 500 µL elution buffer (50 mM K_2_HPO_4_, pH 10), and acidified with 20% TFA, desalted and vacuum-centrifuged to near-dryness.

Total peptides or enriched phosphopeptides were resuspended in 200 mM HEPES (pH 8.5) buffer. Approximately 100 µg total peptides or 50 µg phosphopeptides for each sample were used for proteomic or phosphoproteomic studies, respectively. Peptide samples were labelled with tandem mass tag (TMT) isobaric reagents (Thermo Fisher Scientific) for 1 h and quenched with hydroxylamine, then combined into one tube and acidified with 20% TFA, desalted and vacuum-centrifuged to near-dryness. TMT-labeled sample was resuspended in 400 µL buffer A (1% ACN, 10 mM ammonium formate, pH 9.5) for HPLC fractionation. HPLC fractions were acidified with 20% TFA and vacuum centrifuged to near-dryness, desalted via StageTips and dried by vacuum centrifugation.

### Mass spectrometry data acquisition and processing

Mass spectrometry data were collected as previously described with modifications^24,26,27,56,57^. MultiNotch synchronous precursor selection MS3-based TMT method was used on Orbitrap-Fusion mass spectrometer platform coupled with EASY-nLC liquid chromatography (LC) pump (Thermo Fisher Scientific). Custom made pre-column and analytical column were used for sample trapping and analytical separation. Peptides were separated at a flow rate of 300 nL/min using a gradient of 6%–27% ACN (0.1% formic acid) over 190 min. MS1 spectra (400–1500 m/z) data were acquired at 120,000 resolutions from Orbitrap with 4e5 AGC target, and 50 ms maximum injection time in profile data type. MS1 ions were isolated using the quadrupole with a 0.7 m/z isolation window in top speed mode. MS2 scans (400–1200 m/z) were acquired from the Ion Trap in Turbo mode with CID fragmentation (35% collision energy), 1e4 AGC target, and 50 ms maximum injection time in centroid data type. For TMT reporter ions quantitation, top ten MS2 fragment ions were selected using synchronous precursor selection mode. MS3 scans were acquired from the Orbitrap at 60,000 resolutions with HCD fragmentation (65% collision energy), 1e5 AGC target, and 120 ms maximum injection time in profile data type. Ions were not accumulated for all parallelisable time.

The data of cerebellum and S565A mouse brain samples were collected with the Ultimate 3000 system and Thermo Orbitrap exploris 480 mass spectrometer. The TMT-labeled peptides were separated by using a 120 min gradient elution at a flow rate 0.3 mL/min with the Ultimate 3000 system which was directly interfaced with a Thermo Orbitrap Exploris 480 mass spectrometer. The analytical column was a fused-silica capillary (75 mm ID, 150 mm length; Upchurch, Oak Harbor, WA) packed with C-18 resin (300 A˚, 5 mm; Varian, Lexington, MA). Mobile phase A consisted of 0.1% formic acid, and mobile phase B consisted of 100% acetonitrile and 0.1% formic acid. The Orbitrap Exploris 480 mass spectrometer was operated in the data-dependent acquisition mode using Xcalibur4.5 software. There was a single full-scan mass spectrum in the Orbitrap (300–1800 m/z, 60,000 resolution) followed by 2 s data-dependent MS/MS scans in an ion routing multipole at 36% normalized collision energy.

Raw mass spectrometry files were searched by Proteome Discoverer software (PD, version 2.5, Thermo Fisher Scientific) against a composite target/decoy database using SEQUEST^58–60^. Total 5 raw datasets for SD6 and *Slp* models from Wang et al.^24^. were reanalyzed using same parameters for cross-reference analysis. Protein entries including all isoforms were retrieved from UniProt as the target mouse protein database (April 17, 2022; 94,765). MS2 spectra were searched with fully tryptic restriction, three maximal missed cleavages, ±10 ppm for precursor ion mass tolerance, ±0.8 for fragment ion mass tolerance, fixed TMT modifications on the N terminus and lysine ( + 229.1629 Da) and carbamidomethylation of cysteine residues ( + 57.0215 Da), and dynamic mass shift for oxidation of methionine ( + 15.9949 Da), phosphorylation on serine, threonine and tyrosine ( + 79.9663 Da). The peptide spectrum matches (PSMs) were filtered to achieve 1% protein and peptide FDR (according to *Q* value) by Percolator^61^. Phosphorylation sites were localized by ptmRS51, and phosphopeptides with the phosphorylation site probability score ≥ 25 were used for following analysis. TMT reporter ion signal-to-noise (S/N) values were quantified from MS3 scans with the most confident centroid setting for matching peptides using an integration tolerance of 20 ppm (Orbitrap). The sum of raw reporter ion values for each TMT channel was normalized by assuming equal amount protein input of all channels for following data analyses^24^.

### Mass spectrometry data analysis

Protein isoform was considered as a different protein for proteomic analysis. Phosphopeptide was used for phosphoproteomic analysis including unique and composite (contain ≥ 2 phosphorylation sites) forms. The normalized abundance data (consuming the protein level over each TMT channel was identical) from an experiment were consolidated (sum of value) to generate unique protein or phosphopeptide ID and consolidated abundance values, which were then scaled (the average abundance is one for each subject) to minimize batch effects between different proteomic experiments. The scaled data from different experiments were integrated together based on the unique subject ID for the same comparison (e.g., PD/PS). Then the mean value for each experimental group was used to generate the “log_2_(fold change)” value; and statistical significance (*Q* < 0.2) was determined by the multiple unpaired t-test (*P* value) analysis following the two-stage step-up FDR (*Q* value) approach^62^ unless otherwise stated. Notably, no further filter or computation was performed to high-missing peptides before statistical analysis. The full description and datasets for all phosphoproteomic experiments were listed in Supplementary Table S2, and those for all proteomic experiments were listed in Supplementary Table S3.

The phospho-state change (∆Ps) value was calculated as previously described^24^, which is equal to the “sum of log_2_(fold change) value” of all statistically significant changed (*Q* < 0.2) phosphopeptides from all protein isoforms encoded by the same gene. If none of phosphopeptide’s *Q* value is less than 0.2, the ∆Ps value will be zero. The ∆Ps value for each comparison of brain phosphoproteome was normalized with the total quantified phosphopeptides number of SD6/RS3 comparison for cross-experiment analysis. Due to only few phosphopeptides passed the FDR test (*Q* < 0.2) in RP4/PS comparison, *P* value (*P* < 0.022, equal to *Q* < 0.2 in RP0/PS) was used to determine statistical significance and ∆Ps analysis for RP4/RP0 comparison. A stringent cutoff for ∆Ps value (+/-2.4) was determined by null-tests and applied to identify hyper-phosphorylated (Hyper: ∆Ps>2.4) and hypo-phosphorylated (Hypo: ∆Ps < −2.4) proteins as described12 to represent the concept of cumulative phosphorylation. The full description and datasets for ∆Ps analysis were listed in Supplementary Table S5.

GO cellular component enrichment analysis through Gene Ontology Consortium and PANTHER classification system^63^. KEGG analysis was performed with KEGG Mapper (https://www.genome.jp/kegg) after converting all candidates Uniprot accession to KEGG genes. All 21,997 genes of Mus musculus in database were used as reference to determine the fold of enrichment. Fisher’s Exact with FDR multiple test correction was used to determine statistical significance. A protein will be considered as a synaptic protein when the Synaptic Ref Count ≥ 2 based on the synaptic protein database integrated from 11 previous proteomic studies^24^, gene names were updated according to the UniProt mouse protein database (April 17, 2022; 94,765). Cluster 3.056 was used for hierarchical clustering (Centroid linkage with Euclidean distance). The full datasets for gene ontology analysis were listed in Supplementary Table S4 and synaptic protein database were listed in Supplementary Table S6.

### Kinase motif-enrichment and correlation analysis

Kinase enrichment was performed with previously reported tool (https://kinase-library.phosphosite.org/ea)^35^. In detail, all detected phosphorylation site of each phosphopeptide were represented as site sequence with central position format, which means the phosphorylation site is at the central position of site sequence and the site sequence can include any of the 20 amino acids, ‘X’ for masking a position, and ‘_‘ for truncation. For foreground set definition, *P* value threshold was set to 0.05 for collected 20 experiments, and more stringent *Q* value (0.2) for sleep related phosphoproteome data. To assure foreground set consist of at least 75% of significantly changed phosphopeptides, the log fold change threshold was different from each data due to log fold change range difference. Statistical significance of kinase enrichment analysis was determined using one-sided Fisher’s exact tests, and dominant *P* value was set to 0.01. The full datasets of kinase motif-enrichment analysis were listed in Supplementary Table S7.

Functional Quadrant Enrichment Analysis (FQEA) was performed to every 2 of the 25 comparisons. The kinase enrichment result of each comparison was filter with 0.01 on dominant *P* value. All overlapped kinases of 2 comparisons were plotted on a cartesian coordinate system with dominate log_2_-transformed frequency factor value (dominant_enrichment_value_log_2_) as x or y coordinate. Then, the kinase numbers of 1st or 3rd quadrant (positive correlation direction) and 2nd or 4th quadrant (negative correlation direction) were used to compute *P* value of Chi-square test. Noticeably, the background kinase number of each quadrant was calculated by plotting 303 kinases of each comparison pairs on a coordinate. The FQEA enrichment factor of 2 kinds of correlation directions was the quotient of the corresponding kinase percentage of significantly changed set and background set. For each comparison pairs, the enriched directions were selected based on Chi-square *P* value (0.01) and FQEA enrichment factor.

### PSSM search

A set of reported phosphatase-binding peptides (PBP) for PP1 (RVxF-binding pocket), PP2A (LxxIxE-binding pocket) and PP2B (LxvP and PxIxI-binding pocket) were collected^47,48^, which consist of 107 RVxF, 25 LxxIxE, 34 LxvP and 33 PxIxI motifs. A position-specific scoring matrix (PSSM) was constructed from the aligned sequences using the PSI BLAST IC scoring scheme with PSSMSearch tool. Then, each PSSM was searched against the mouse proteome respectively and the hits were filtered based on the PSSM score *P* value with a cut-off of 0.0001 and intrinsic disorder regions which IUPred score is less than 0.3. Applying these criteria, we produced 1903 putative phosphatase-binding motifs on 1575 proteins and performed following motif analysis on phosphoproteomics data with them. The full datasets of PSSM search were listed in Supplementary Table S9.

### Plasmids construction

Mouse open reading frame (ORF) of *sSLP* (*Sik3* A60-K527, NM_027498.3) was synthesized from Comate Bioscience. *SV40* (small T antigen, GenBank: LT727623.1) and human *LCMT1* (20-338, NM_001032391.2) ORF were synthesized from Miaoling Biology. Mouse *Ppp2ca* and *Ppp2r5d* ORF was purchased from Miaoling Biology (P34019 and P21643, respectively). *Camk2a*, *mDlx*, *GfaABC1D* promoters were from *pAAV-CaMKIIa-hM4D(Gi)-mCherry* (Addgene, 50477), pAAV-*mDlx*-GFP-Fishell-1 (Addgene, 83900) and pAAV-*GfaABC1D*-GRAB_Ado1.0 (Addgene, 153288). A221 (*sSLP* T221A) mutant was constructed using QuickMutation Site-Directed Mutagenesis Kit (Beyotime, D0206). *sSLP*, *A221*, *SV40*, *LCMT1*, *Ppp2ca* and *Ppp2r5d* were subcloned into pAAV-*hSyn*-EGFP (Addgene, 50465) by replacing EGFP. *Camk2a*, *mDlx*, and *GfaABC1D* promoters were subcloned into pAAV-*hSyn*-sSLP or pAAV-*hSyn*-SV40 by replacing *hSyn*.

### Virus production and purification

AAVs packaging and purification was performed as previously described with modifications^46,64,65^. AAVs were generated in the AAVpro 293 T cell line (Clontech, 632273) using polyethylenimine “Max” (PEI MAX, Polysciences) with pAAV-PHP.eB, pHelper and pAAV/*hSyn*-target gene expressing plasmids. The cell line was not tested for mycoplasma. The culture medium containing the transfection reagent was replaced with serum-free DMEM at 12–15 h post-transfection. Recombinant AAVs was collected at 6 days post-transfection. The cell and medium mixture were centrifuged at 17,940 *g* for 10 min at room temperature. Cell pellets were resuspended in 1 mL phosphate buffered saline (PBS), freeze-thawed for five times, and centrifuged at 17,940 *g* for 20 min. The supernatant was combined with cleared virus containing medium, and then filtered with 0.45 μM sterile filter. Viral media was concentrated using the Vivaspin 20 column (Cytiva) and formulated with PBS twice. The concentrated viral solution was stored at −80 °C until systemic administration. Virus titers were measured using a linearized genome plasmid as a standard. Intravenous administration of 100 μl AAV viral solution (1.0×10^12^ vg/mL) was performed by injecting the virus into the retro-orbital sinus under deep anesthesia by isoflurane.

### Brain slice preparation and electrophysiology

For all electrophysiology experiments, brain slices were prepared from 12 to 16 weeks old mice. The motor cortex slice preparation for electrophysiological recordings followed previous neocortical studies^66,67^. Briefly, brains were rapidly removed after decapitation and placed into ice-cold (0–4 °C) artificial cerebrospinal fluid (ACSF) solution containing 119 mM NaCl, 2.5 mM KCl, 1 mM NaH_2_PO_4_, 26 mM NaHCO_3_, 1 mM MgCl_2_, 25 mM glucose and 2 mM CaCl_2_, pH 7.4 with NaOH, osmolarity 300–305 mOsm saturated with 95% O_2_ and 5% CO_2_. The brain was then placed on a custom ramp with a 5° tilt angle and slices (400 μM) were cut with a vibratome (VT1200S, Leica Biosystems) coronally to retain axonal and dendritic arborizations of neurons as intact as possible. The brain slices were kept at 37.0 °C in oxygenated ACSF solution for 20 min, then transferred to the recording chamber.

All recordings were obtained from the layer 5 of the motor cortex pyramidal neurons. Recording pipettes were pulled from borosilicate to resistances of 4–7 MΩ with a Sutter Instruments P-97. Micropipettes were filled with an intracellular solution containing the following: 120 mM potassium gluconate, 10 mM HEPES, 4 mM KCl, 4 mM MgATP, 0.3 mM Na_3_GTP, 10 mM sodium phosphocreatine (pH 7.25), osmolarity 300 mOsm/kg. Motorized micromanipulators (Junior Multpatch, Lugis & Neumann) were used to steer pipette electrodes to target cells. Whole cell recordings were made with Model 2400 amplifiers (A-M Systems). Electrophysiological data acquisition and stimulation command sending were performed by synchronized LIH 8 + 8 data acquisition interface boards (HEKA Instruments), which were operated by a custom-written Igor Pro 6 program.

### Immunoblotting

Western blotting was performed according to standard procedures using the corresponding antibodies. Antibodies were used at the optimal concentration according to the manufacturer’s instructions. Antibodies used in this study included anti-phospho-AMPK Substrate Motif (LXRXX(S*/T*)) (#5759, Cell Signaling), anti-phospho-PKC substrate motif ((K/R)XS*X(K/R)) (#6967, Cell Signaling), anti-phospho-ATM/ATR substrate motif (S*Q) (#9607, Cell Signaling) and anti-HA (C29F4) (#3724, Cell Signaling), p-HDAC4 S246 (#ab240643, Abcam), p-KCC2 S940 (#612-401-E15, Rockland), p-nNOS S1417 (#ab5583, Abcam), p-eEF2 T56/T58 (#ab82981, Abcam) and p-PRKAR1A S77 (#ab139682, Abcam).

### Statistical analysis

Unless otherwise noted, all experimental subjects were biological replicates and at least two independent experiments were performed. Mice were randomly assigned to groups. Researchers were blinded to the treatments during EEG/EMG data manual correction. No statistical methods were used to predetermine sample size. GraphPad Prism, EXCEL or Python software was used for statistical test analysis. Following one-way or two-way analysis of variance (ANOVA), Tukey’s test compares every mean with every other mean; Dunnett’s test compares every mean to a control mean; Sidak’s test compares a set of means. Repeated measures (RM) or paired tests were performed for matched subject comparisons. The complete sample size, statistical test method and results for each comparison are reported in each figure legend and fully described in Supplementary Table S10.

## Supplementary information

Supplementary information

Dataset 1

Dataset 2

Dataset 3

Dataset 4

Dataset 5

Dataset 6

Dataset 7

Dataset 8

Dataset 9

Dataset 10

## Data Availability

The mass spectrometry datasets include raw data files, search engine files and a full experimental summary file have been deposited to MassIVE database: MSV000089587.
