# Supplementary material for: Sleep prevents brain phosphoproteome disruption to safeguard survival
Source: Cell Discov. 2025 Jun 24;11:58. doi: 10.1038/s41421-025-00809-w (PMC12187926; doi:10.1038/s41421-025-00809-w)
Supplement: Supplementary file 1 — Supplementary information [file 41421_2025_809_MOESM1_ESM.pdf]

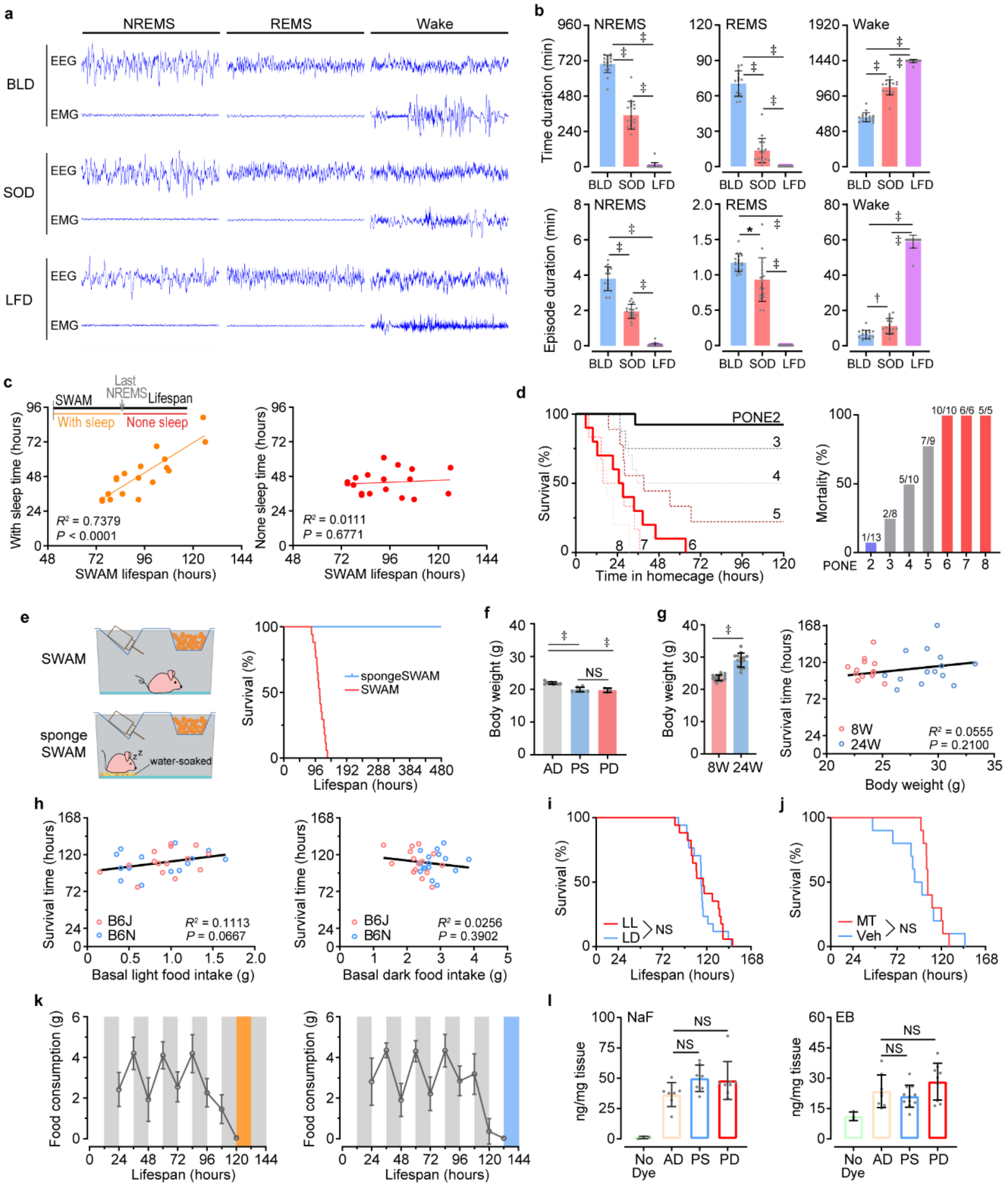

Fig. S1 Sleep and confusing factors analysis of the SWAM model.

a Representative 8-s EEG and EMG for NREMS, REMS and wake from baseline day (BLD), SWAM onset day (SOD), last full day (LFD) for C57BL/6J mice. **b** Quantitative analysis of total time (*top*) and mean epoch time (*bottom*) of NREMS, REMS and wake states on BLD, SOD and LFD (n = 18 per group). **c** Person correlation between survival time and with-sleep (*left*) and none-sleep period (*right*) (n = 18 per group). **d** The survival (*left*) and mortality (*right*) rate of mice with different PONE index value. The number on each column represents [death/total]. **e** A schematic of the sponge SWAM experiment (*left*), and the survival analysis of SWAM with (n = 12) and without sponge (n = 17) (*right*). **f**

The body weight of PS and PD mice. **g** Body weight (*left*) and Person correlation (*right*) of 7 (n = 16) and 24 (n = 14) weeks B6J mice. **h** Pearson correlation between survival time and basal light (*left*) or dark (*right*) phase food intake. **i, j** Survival analysis of C57BL/6J mice in constant light (LL, n = 17) and regular light/dark (LD, n = 17) conditions (**i**), vehicle (Veh, n = 10) and melatonin (MT, n = 10) injected subjects (**j**). **k** Circadian analysis of daily food consumption during SWAM for mice dead at light phase (*left*, n = 9) or dark phase (*right*, n = 5), each data point represents the mean value in the following light or dark phase. **l** Evaluation of blood-brain barrier integrity using sodium fluorescein (NaF, *left*; n = 3 (No dye), 7 (AD), 7 (PS), 8 (PD)) and Evans blue (EB, *right*; n = 3 (No dye), 8 (AD), 11 (PS), 9 (PD)). Data are mean ± s.d. One-way ANOVA with Sidak's test (**b** *top*); two-tailed paired t-test (**b** *bottom*); Log-rank (Mantel-Cox) test (**e**, **i**, **g**); unpaired t-test (**f**, **g**, **l**). * $P < 0.05$; † $P < 0.01$; ‡ $P < 0.001$; NS, not significant, $P > 0.05$. n refers to the number of biological replicates.

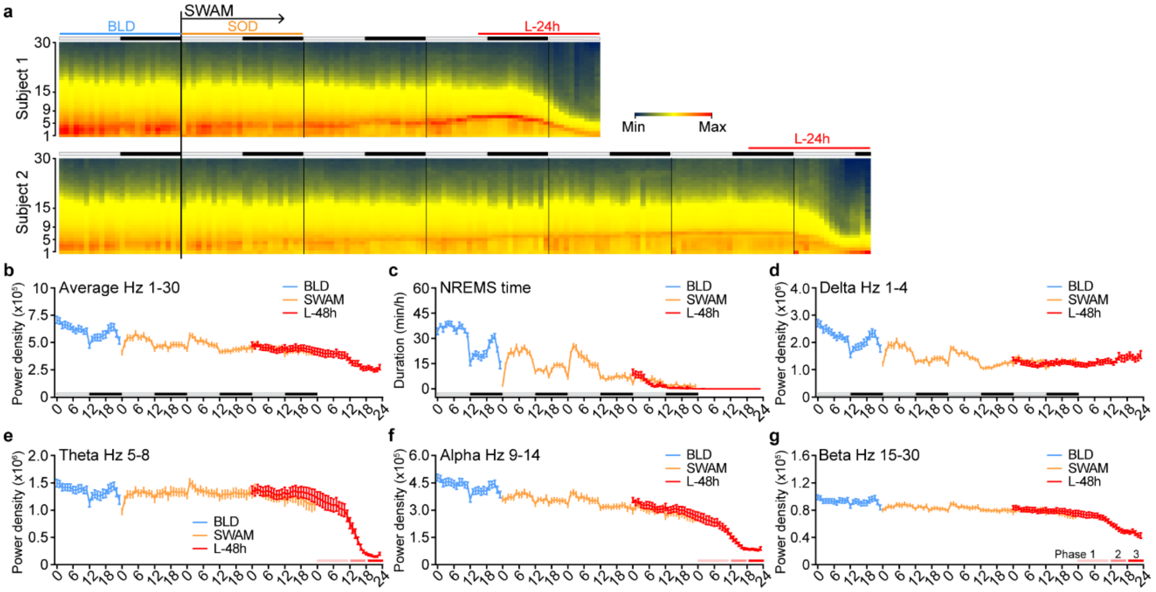

Fig. S2 Sleep analysis of SWAM model.

a Heat map plot of absolute EEG power of 2 subjects. **b-g** The analysis of total absolute EEG power (not distinguish sleep or wake status) of average (**b**), Delta (**d**), Theta (**e**), Alpha (**f**) and Beta (**g**) band, and duration of NREMS (**c**) during SWAM (n = 15). * $P < 0.05$; † $P < 0.01$; ‡ $P < 0.001$; NS, not significant, $P > 0.05$. n refers to the number of biological replicates.

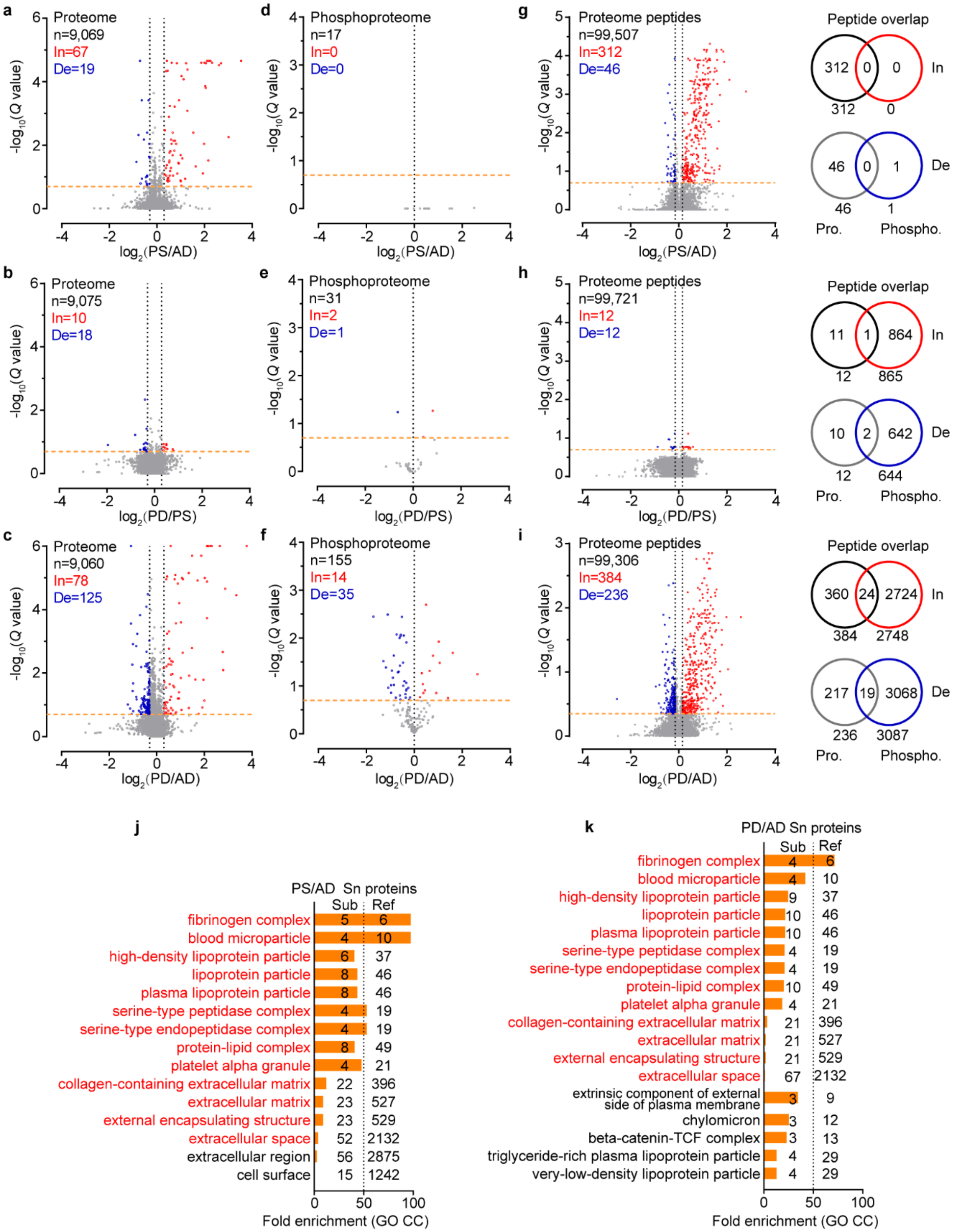

Fig. S3 Brain proteomic analysis of the Pr-SD model.
a-c Volcano plots showing comparative analysis of PS/AD (**a**), PD/PS (**b**) and PD/AD (**c**) proteomes. Multiple unpaired t-test (*P* value) following FDR (*Q* value) analysis. X-axis, $\log_2$(fold change) in abundance; Y-axis, $-\log_{10}(Q$ value) of abundance change. The numbers of total (n), increased [In: $Q < 0.2$, $\log_2 > 0.3$, red] and decreased [De: $Q < 0.2$, $\log_2 < -0.3$, blue] subjects are shown. Orange dotted lines ($Q = 0.2$). **d-f** Volcano plots

displaying phosphopeptides of those significantly changed proteins in PS/AD (**d**), PD/PS (**e**) and PD/AD (**f**) proteomes. In, increased; De, decreased. Multiple unpaired t-test (*P* value) followed by false discovery rate (FDR) (*Q* value) analysis. **g-i** Volcano plots showing comparative analysis (*left*) of PS/AD (**g**), PD/PS (**h**) and PD/AD (**i**) proteome peptides, and significantly changed peptides overlap between proteome and phosphoproteome (*right*). **j, k** Gene ontology cellular component (GO CC) enrichment analysis of those significantly changed protein subjects (Sub) in PS/AD (**j**) and PD/AD (**k**) comparisons. Ref: the number of reference gene in corresponding GO items. Fisher's Exact with FDR multiple test correction was used to determine statistical significance using all 21,997 genes of Mus musculus as reference (Ref). GO terms (FDR < 0.05, enrichment > 3), the gene number of Sub and Ref in each term are shown.

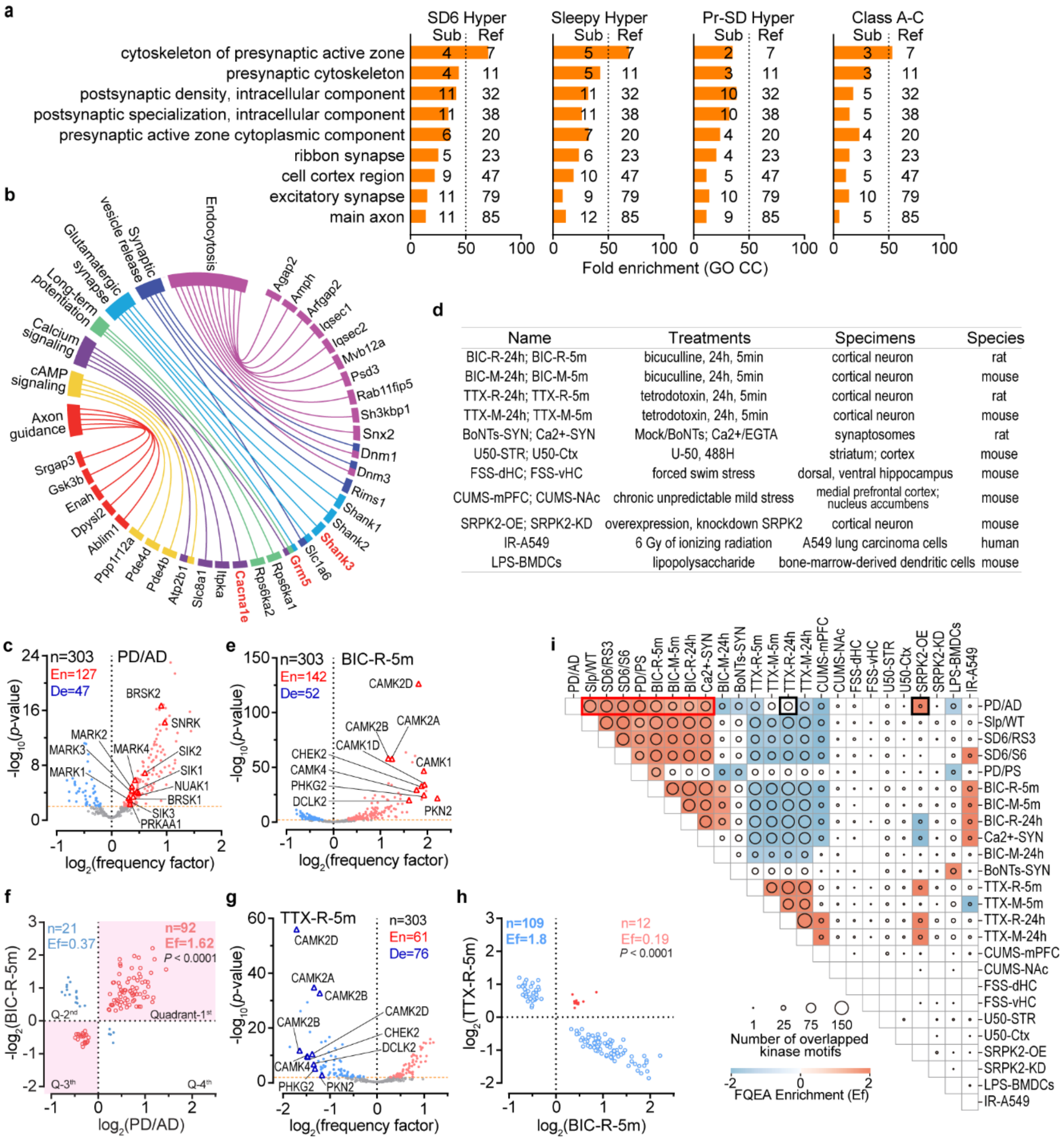

Fig. S4 Bioinformatic analysis of SWAM phosphoproteome.

a The top overlapped GO terms of Hyper proteins in SD6, Sleepy and Pr-SD models. **b** Phosphoproteins (class A-C and PD-SNIPPs) assigned to representative KEGG pathways. **c** Global kinase motif enrichment analysis of phosphoproteomic data from PD/AD comparison. **d** The information of collected phosphoproteomic datasets. **e** Global kinase motif enrichment analysis of phosphoproteomic data from BIC-R-5m. **f** FQEA analysis between PD/AD and BIC-R-5m. **g** Global kinase motif enrichment analysis of phosphoproteomic data from TTX-R-5m. **h** FQEA analysis between PD/AD and TTX-R-5m. **i** FQEA analysis matrix for all 25 comparisons. The dot size represents the number of overlapped kinase motifs and dot color shows enrichment factors. two-sided Chi-square (**f**, **h**).

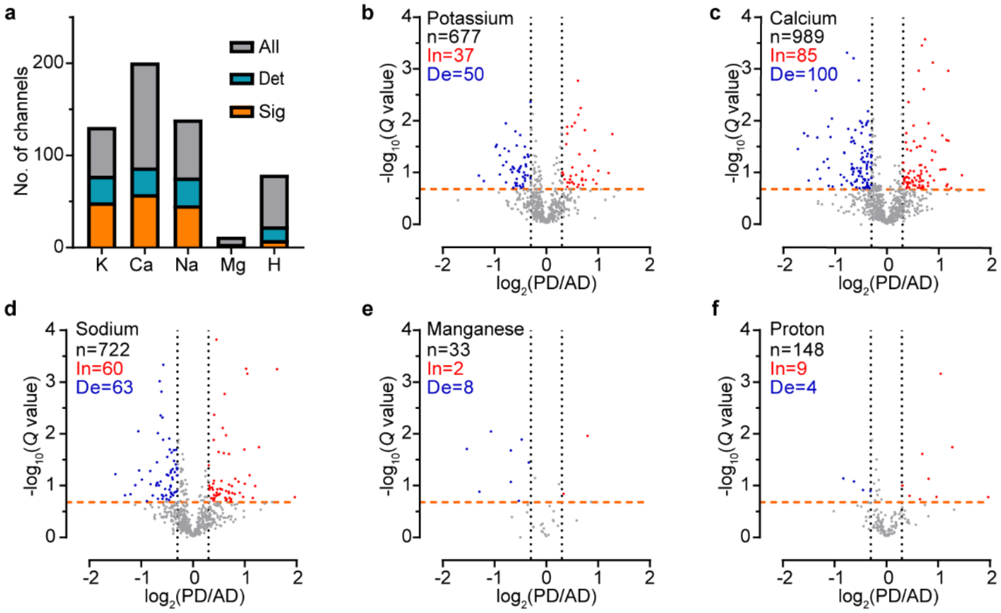

Fig. S5 Phosphoproteomic analysis of ion channel proteins.

a The number of proteins in each ion channel categories. All: all ion channel proteins; Det: Detected proteins in PD/AD group; Sig: Protein with significantly changed phosphopeptides. **b-f** Volcano plots showing comparative analysis of potassium (**b**), calcium (**c**), sodium(**d**), manganese (**e**) and proton (**f**) ion channels phosphopeptides. Multiple unpaired t-test (*P* value) following FDR (*Q* value) analysis. X-axis, $\log_2$(fold change) in abundance; Y-axis, $-\log_{10}$(*Q* value) of abundance change. The numbers of total (n), increased and decreased subjects are shown. Orange dotted lines ($Q = 0.2$).

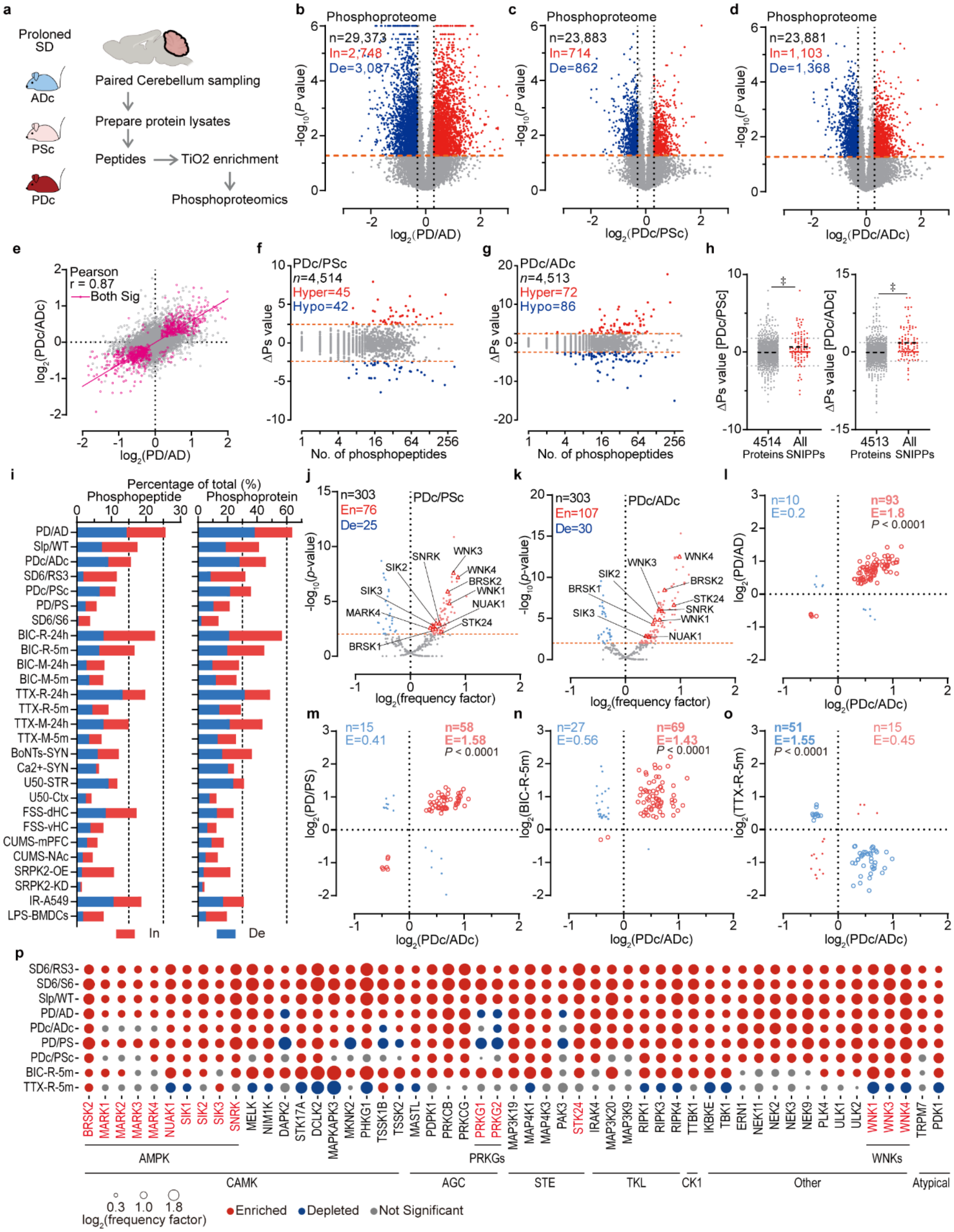

Fig. S6 Phosphoproteomic analysis of cerebellum in Pr-SD model.

a Experimental design for quantitative phosphoproteomic studies of cerebellum from SWAM treated mice. **b-d** Volcano plots displaying changed phosphopeptides in PD/AD (**b**), PDc/PSc (**c**) and PDc/ADc (**d**) groups. Orange dotted lines (P = 0.054, equal to Q = 0.2 in PD/AD). **e** Scatter plot of peptides occurred both in PD/AD and PDc/ADc groups. **f-h** Global ΔPs analysis of phosphoproteins in PDc/PSc (**f**) and PDc/ADc (**g**) groups, and

quantitative ΔPs analysis of SNIPPs in PDc/PSc (**h**, *left*) and PDc/ADc (**h**, *right*) comparisons. Dotted lines represent ΔPs = ±2.4. Numbers of total, hyperphosphorylated (Hyper) and hypophosphorylated (Hypo) proteins are shown. **i** Systematic analysis the percentage of significantly changed phosphopeptides (**i**, *left*) and phosphoproteins (**i**, *right*) among 27 comparisons. **j, k** Global motif enrichment analysis of phosphoproteomic data from PDc/PSc (**j**) and PDc/ADc (**k**) comparisons. En, enriched; De, depleted. **l-o** FQEA analysis of PDc/ADc with PD/AD (**l**), PD/PS (**m**), BIC-R-5m (**n**) and TTX-R-5m (**o**) comparisons. **p** Frequency factor for each kinase in different comparisons. Two-tailed unpaired t-test (**h**); two-sided Chi-square (**l-o**). * $P < 0.05$; † $P < 0.01$; ‡ $P < 0.001$.

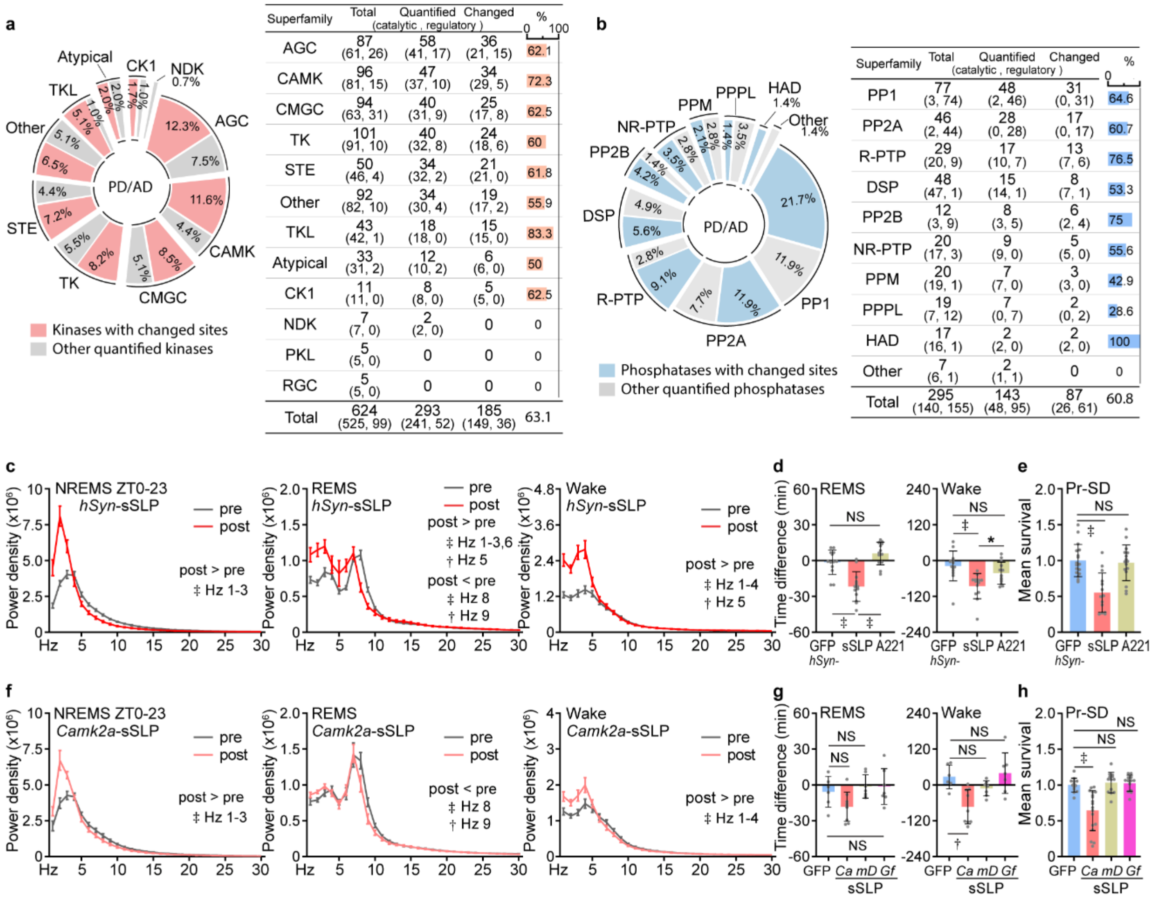

Fig. S7 Sleep analysis of SLEEPY expression mice.

a, b The distribution of kinases (**a**) and phosphatases (**b**) with changed phosphopeptides among different superfamily (*left*), and a detailed summary for the mouse kinome or phosphatome changes (*right*) in PD/AD comparison. **c, d** Absolute EEG power spectra (**c**) and time difference (**d**) of NREMS, REMS or wake in mice injected with *hSyn*-GFP, *hSyn*-A221 and *hSyn*-sSLP (n = 13 per group). **e** The individual survival of mice injected with *hSyn*-GFP (n = 17), *hSyn*-A221 (n = 16) and *hSyn*-sSLP (n = 14). **f, g** Absolute EEG power spectra (**f**) and time difference (**g**) of NREMS, REMS and wake in mice injected with *hSyn*-GFP (n = 6), *Camk2a*-sSLP (n = 9), *mDlx*-sSLP (n = 7) and *GfaABC1D*-sSLP (n = 7). **h** The individual survival of mice injected with *hSyn*-GFP (n = 13), *Camk2a*-sSLP (n = 18), *mDlx*-sSLP (n = 13) and *GfaABC1D*-sSLP (n = 13). Data are mean ± s.d. Two-way ANOVA with Sidak's test (**c**, **f**); one-way ANOVA with Tukey's test (**d**, **e**); one-way ANOVA with Dunnett's test (**g**, **h**). * $P < 0.05$; † $P < 0.01$; ‡ $P < 0.001$; NS, not significant, $P > 0.05$. n refers to the number of biological replicates.

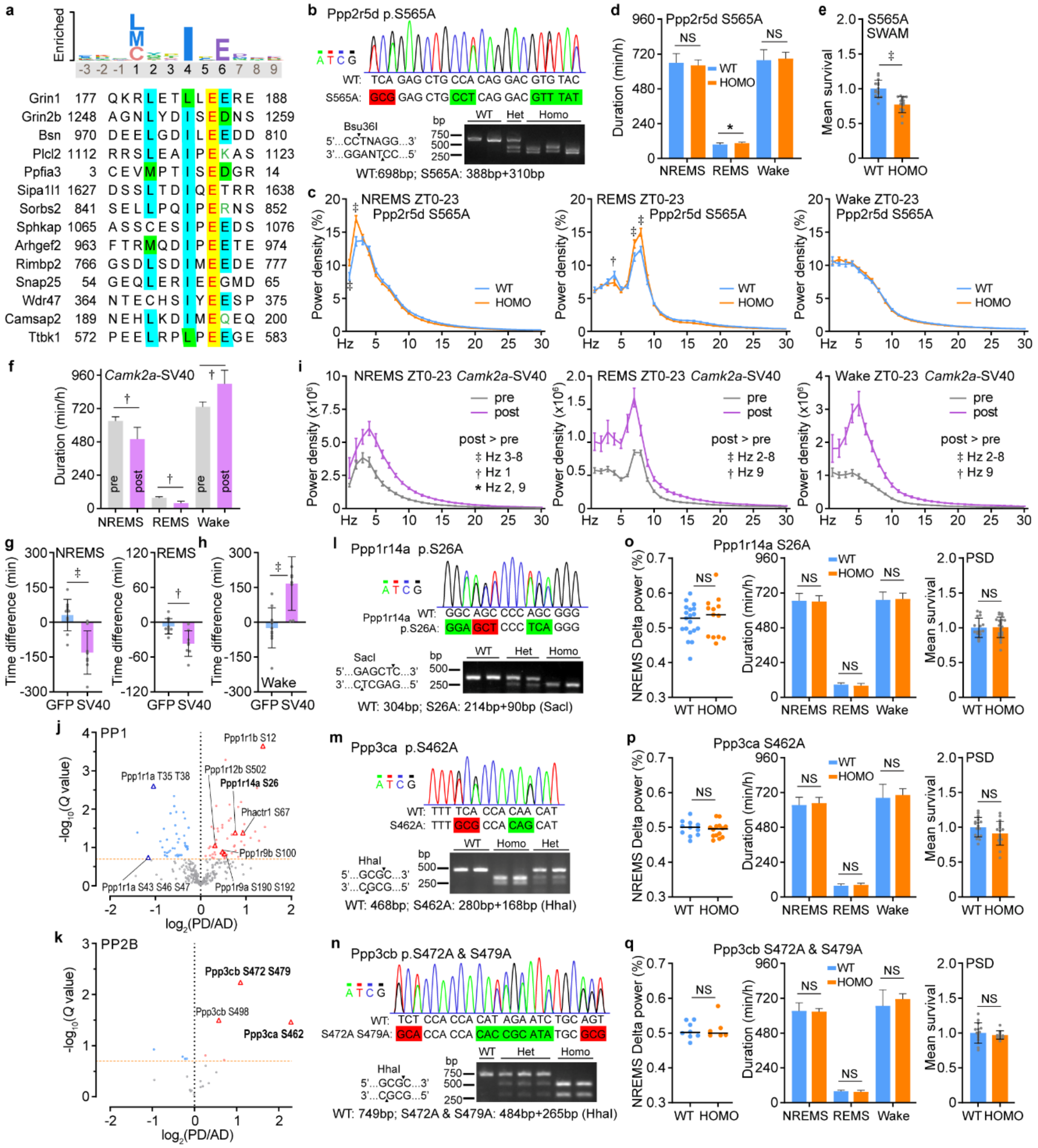

Fig. S8 Sleep and Pr-SD analysis of phosphatase-related gene manipulation mice.
a Alignment the LxxIxE motifs from putative PP2A substrates. **b** Direct sequencing, genotyping designs, and RT-PCR of the *Ppp2r5d* (S565A) mutant mouse. **c, d** Absolute EEG power spectra (**c**) and duration (**d**) of NREMS, REMS and wake in of *Ppp2r5d* (S565A) homozygous (HOMO, n = 14) and wild-type (WT, n = 13) littermates. **e** The individual survival of *Ppp2r5d* (S565A) HOMO and WT (n = 15 per group) littermates. **f-i** The duration (**f**), time difference (**g**, **h**) and absolute EEG power spectra (**i**) of NREMS, REMS and wake in mice injected with *Camk2a*-SV40 (n = 8) and *Camk2a*-GFP (n = 10). **j, k** Volcano plots of quantified phosphopeptides of PP1 (**j**) and PP2B (**k**) family in PD/AD comparison. **l-n** Direct sequencing, genotyping designs, and RT-PCR of the *Ppp1r14a* (S26A) (**l**), *Ppp3ca* (S462A) (**m**) and *Ppp3cb* (S472A/S479A) (**n**) mutant mice. **o-q** Analysis of mean relative NREMS delta power (*left*), duration (*middle*), and individual

survival (*right*) for *Ppp1r14a* (S26A) (**o**, *n* = 19 and 13, WT and HOMO), *Ppp3ca* (S462A) (**p**, *n* = 11 and 14, WT and HOME) and *Ppp3cb* (S472A/479A) (**q**, *n* = 8 and 6, WT and HOMO) homozygous mice and littermates. Data are mean ± s.d. Two-way ANOVA with Sidak's test (**c**, **i**); two-tailed unpaired t-test (**d**, **e**, **g**, **l**, **o**-**q**); two-tailed paired t-test (**f**). * $P < 0.05$; † $P < 0.01$; ‡ $P < 0.001$; NS, not significant, $P > 0.05$. *n* refers to the number of biological replicates.

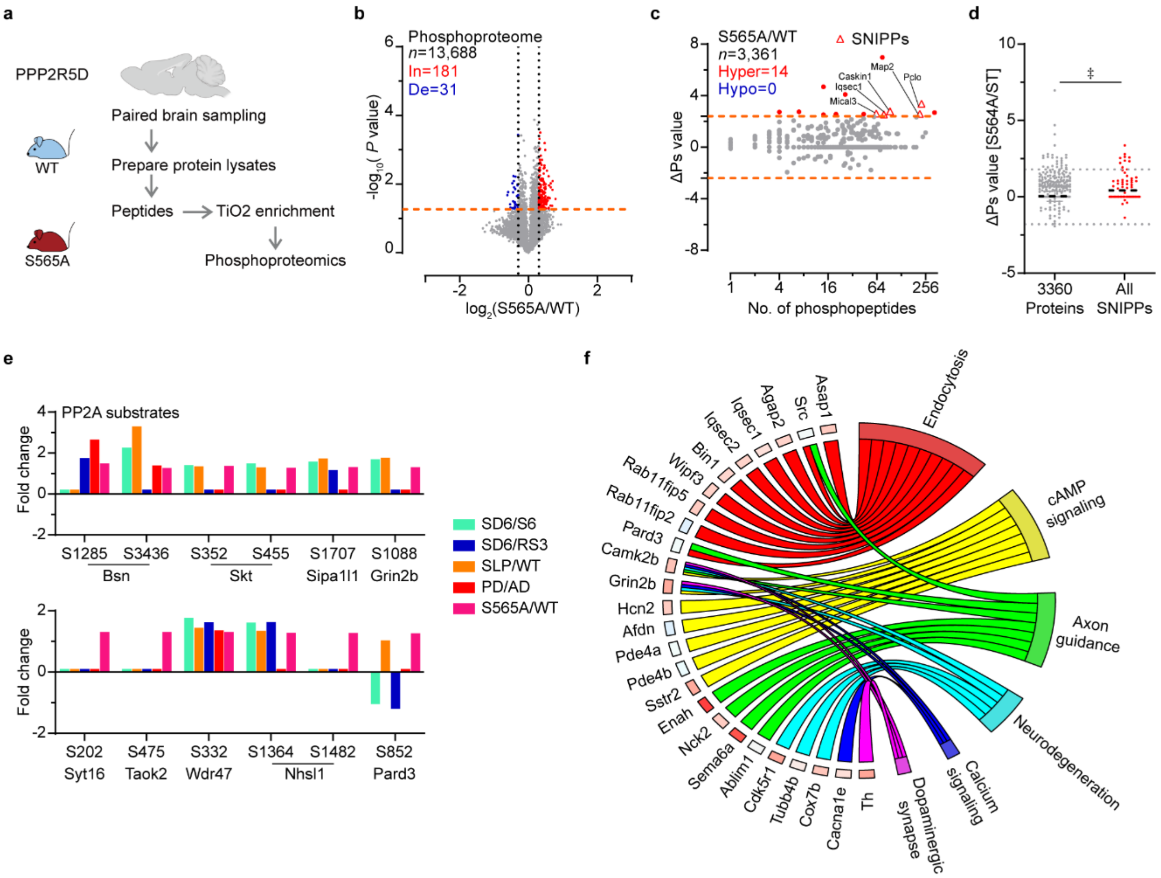

Fig. S9 Phosphoproteomic analysis of PPP2R5D S565A mice.

a Experimental design for quantitative phosphoproteomic studies of PPP2R5D S565A mutant mice. **b** Volcano plots displaying changed phosphopeptides in S565A/WT group. In, increased [$\log_2$(ratio)>0.3]; De, decreased [$\log_2$(ratio)<−0.3]. **c** Global ΔPs analysis of phosphoproteins in S565A/WT group. **d** Quantitative ΔPs analysis of SNIPPs in S565A/WT comparison. **e** Quantification of phosphorylation sites from PP2A substrates. **f** Phosphoproteins with significantly changes peptide assigned to representative KEGG pathways. Two-tailed unpaired t-test (**d**). * $P < 0.05$; † $P < 0.01$; ‡ $P < 0.001$.

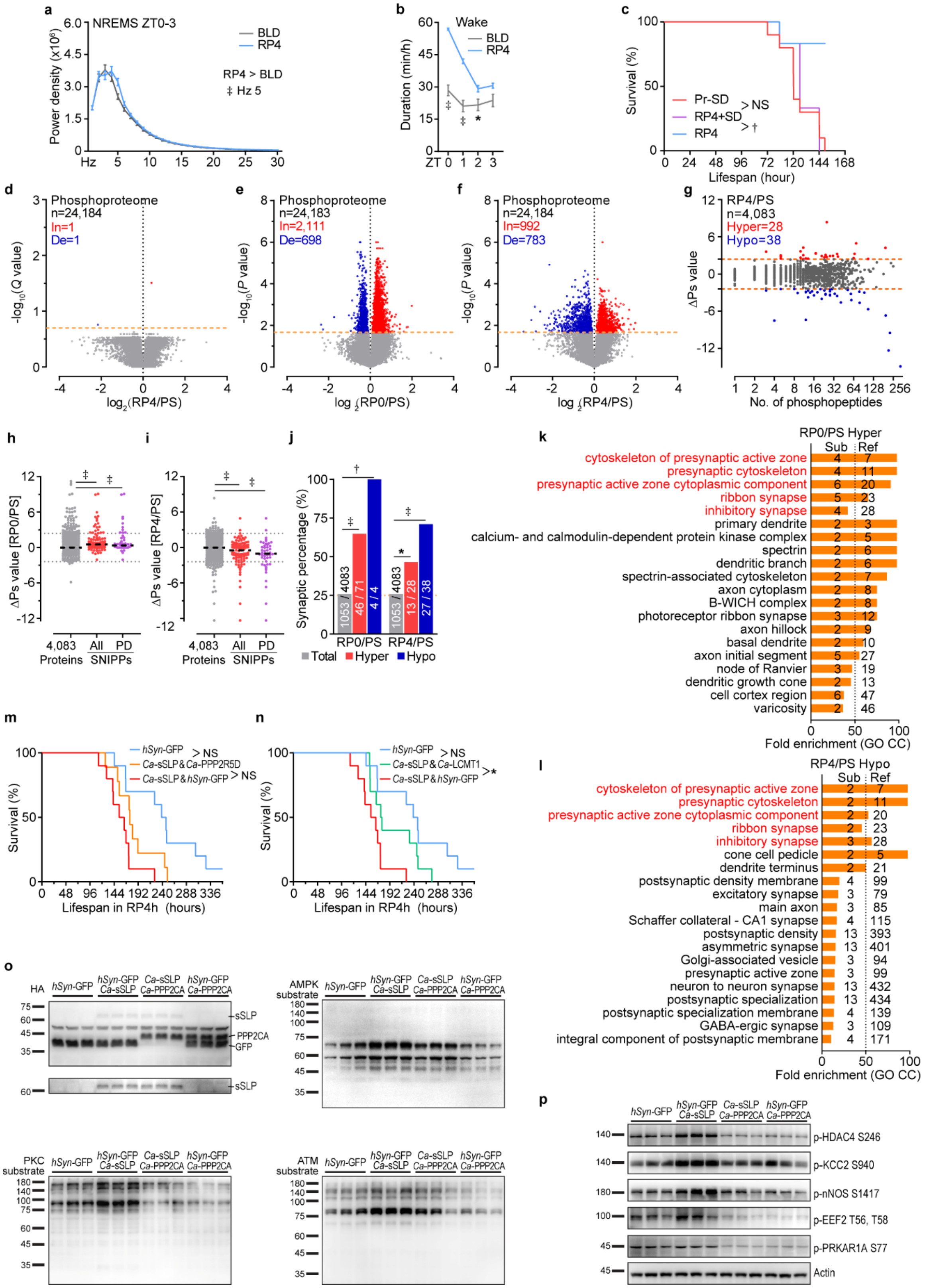

Fig. S10 Brain phosphoproteomic analysis of the RP4h model.

a, b Absolute EEG power spectra of NREMS (**a**), and wake duration (**b**) during RP4h (*n* = 16). **C** Survival analysis of RP4h treatment with or without combining classical sleep

deprivation. **d** Volcano plots displaying changed phosphopeptides in RP4/PS group. Orange dotted lines ($Q = 0.2$). **e, f** Volcano plots displaying changed phosphopeptides in RP0/PS (**e**) and RP4/PS (**f**) group. Orange dotted lines ($P = 0.022$, equal to $Q = 0.2$ in RP0/PS). **g-i** Global ΔPs analysis of phosphoproteins in RP4/PS group (**g**), and quantitative ΔPs analysis of SNIPPs in RP0/PS (**h**), RP4/PS (**i**) comparison. Dotted lines, ΔPs = ±2.4. **j** Percentage of synaptic proteins in total, Hyper- and Hypo-phosphoproteins in RP0/PS and RP4/PS groups. Numbers of synaptic and total phosphoproteins for each group are shown. **k, l** Gene ontology cellular component (GO CC) enrichment analysis of 71-Hyper-proteins in RP0/PS (**k**) and 38-Hypo-proteins in RP4/PS (**l**) comparisons. Top 20 GO terms (FDR < 0.05, enrichment > 3), the gene number of subjects (Sub) and Ref in each term are shown. **m, n** The survival of SWAM mice with RP4h and injected with AAV of PPP2R5D (**m**) and LCMT1 (**n**) (n = 9 (GFP&PPP2R5D), 10 (rest groups)). **o** Immunoblots of AAV infected mice brain with antibody specific for HA tag or AMPK, PKC and ATM target phosphorylation motifs. **p** Immunoblots of AAV infected mice brain with antibody specific for phosphorylation sites of HDAC4 S246, KCC2 S940, nNOS S1417, EEF2 T56/T58 and PRKAR1A S77. Data are mean ± s.e.m. Two-way ANOVA Sidak's test (**a**, **b**); log-rank (Mantel-Cox) test (**c**, **m**, **n**) one-way ANOVA with Tukey's test (**h**, **i**); two-sided Chi-square (**j**). * $P < 0.05$; † $P < 0.01$; ‡ $P < 0.001$; NS, not significant, $P > 0.05$. n refers to the number of biological replicates.

Table S1
Data lists for PONE index analysis. Representative results and analyses of PONE index were listed.

Table S2
Data lists of quantitative phosphoproteomic analysis of whole brain samples. The full description and datasets for all phosphoproteomic experiments were listed. Statistical analysis and *n* numbers for all comparisons were shown.

Table S3
Data lists of quantitative proteomic analysis. The full description and datasets for all proteomic experiments were listed. Statistical analysis and *n* numbers for all comparisons were shown.

Table S4
Data lists for bioinformatic analysis. The full description and gene ontology and KEGG analyses were listed.

Table S5
Data lists of protein phosphorylation state analysis. The full protein phospho-state analysis description and datasets for all comparisons were listed.

Table S6
Database of synaptic, kinome and phosphatome. An integrated synaptic, kinome, phosphatome and ion channels protein databases were listed.

Table S7
Data lists of kinase motif enrichment analysis. The full description and kinase motif enrichment analysis were listed. Statistical analysis and data for all comparisons were shown.

Table S8
Data lists of quantitative phosphoproteomic analysis of cerebellum and S565A mutant samples.

Table S9
Data lists of phosphatase motif analysis. The full description of phosphatase SLiMs and phosphatase motif analysis were shown.

Table S10
Statistical analysis. The complete sample size, statistical test methods and precise value results for all comparisons were reported.

Discussion

1. Selection of PONE Index Cutoffs

The decision to utilize two specific PONE index cutoffs was driven by the necessity to balance theoretical insights, experimental limitations, and the need to capture the dynamic progression of the PONE state effectively. The progression of the PONE index was characterized by a prolonged low-value phase, followed by an acute surge occurring within approximately 12 hours (**Fig. 1h**). Data simulation analysis revealed that this acute elevation typically began around a PONE index of 2 (1.7, based on simulations), representing a critical point where the PONE index began to escalate sharply. This theoretical value was thus identified as the lower cutoff, marking the initial transition towards a potentially irreversible state.

Despite the significance of this transition, practical considerations underscored the challenge of pinpointing this threshold with absolute certainty. Behavioral observations inherently involve fluctuations due to observational deviations, resulting in potential variability in the recorded PONE index (ranging between 0 and 4 near index 2). Consequently, we could not definitively ascertain whether a recorded PONE index of 2 was the terminal or an intermediate value before the mice succumbed to SWAM-induced stress.

To address this uncertainty, we established a second, higher cutoff point to ensure the robust identification of mice firmly within the PONE state. A straightforward candidate was the maximum evaluable PONE index of 8; however, this value was exceedingly rare due to the fixed time intervals of scoring, rendering it impractical for analysis. Instead, we evaluated the remaining survival durations associated with each PONE index (**Fig. 1l**). Notably, PONE indices of 5, 6, and 7 shared a comparable remaining survival time of approximately 6 hours, while indices of 3 and 4 were associated with a survival duration closer to 12 hours. Based on these observations, a cutoff within the range of 5 to 7 was deemed more appropriate, as it better captured the point where the mice were more likely to have entered an irreversible state.

Among these, we selected a PONE index of 6 as the upper cutoff due to its symmetric position relative to index 2 in the reverse countdown of survival progression. Subsequent experiments confirmed the utility of this choice: over 90% of mice with a PONE index $\geqslant$ 6 ultimately perished, whereas mice with a PONE index $\leqslant$ 2 exhibited a mortality rate of just 5.2%. These results provided strong support for the appropriateness of these cutoffs in distinguishing mice at different stages of the PONE state.

It is important to note that while the PONE state may manifest earlier in some instances—mice with a PONE index between 2 and 6 also demonstrated increasing mortality risk—the chosen cutoffs offered a pragmatic framework for investigating the PONE state with greater confidence. Our current behavioral and phosphoproteomic analysis approach provides a robust foundation for studying this phenomenon, although

future research may refine these definitions and identify more precise biomarkers for PONE state characterization.

By defining PONE index 2 as the lower threshold and PONE index 6 as the upper cutoff, we aimed to capture the transition and progression of the PONE state effectively. This approach enables a nuanced understanding of this complex state while maintaining a manageable experimental framework, paving the way for future explorations into its underlying mechanisms.

2. The limitations of PONE index system

The PONE status prediction method used in our study was based solely on behavioral observations, which had the following limitations. Behavioral observations can be subjective and prone to bias, depending on the observer's interpretation of the behaviors. This can lead to inconsistencies in data collection and minor discrepancy of PONE index development in different experiment environment or mouse strain. Behavioral observations are recorded at specific intervals, which may not capture real-time changes. However, after a period of preliminary experiments, a standardized scoring protocol could be established in any lab.

3. Minimal Phosphoproteomic Differences Between PS and AD Groups

The minimal differences in the phosphoproteome between the PS and AD groups observed in this study were indeed surprising, especially given that previous research has demonstrated significant changes in the brain phosphoproteome after sleep deprivation[1]. Several factors may help explain this discrepancy, particularly the nature of the sleep deprivation protocol employed in our study, which differs significantly from classical methods of complete sleep deprivation.

Firstly, our sleep deprivation model—SWAM —does not involve complete sleep deprivation but rather results in intermittent sleep bouts, especially during the early phases of sleep deprivation (**Fig. 1e and Supplementary Fig. S1b**). Unlike the classical sleep deprivation protocols, where animals are subjected to acute, complete sleep loss, SWAM allows for small periods of sleep, even in the initial stages of sleep deprivation. This intermittent sleep could mitigate the extent of phosphoproteomic changes typically observed under conditions of complete sleep deprivation. Specifically, the phosphoproteomic analysis under the RP4h conditions in this study confirmed that even after prolonged sleep loss, the brain's phosphoproteome exhibits partial recovery following short periods of sleep (i.e., a brief recovery sleep after sleep deprivation). These results are consistent with our previous findings in the SD6 protocol, where a short recovery sleep significantly reversed the phosphoproteomic alterations induced by sleep deprivation.

The intermittent nature of sleep deprivation during the Pr-SD phase could, therefore, account for the relatively small differences between the PS and AD groups. Unlike the complete and sustained sleep deprivation used in earlier studies, which would lead to more pronounced changes in the phosphoproteome, the SWAM model's intermittent sleep bouts likely buffer against the full extent of such alterations. This may explain why the phosphoproteomic differences between PS and AD groups were more subtle than anticipated.

In addition to the intermittent sleep bouts, the sleep homeostasis mechanisms may also play a role in the observed minimal differences. Previous research by Franken et al. suggested that NREMS delta power, a key marker of sleep pressure, exhibited a smaller than expected increment following extended periods of sleep deprivation (beyond 9 hours)[2]. This could indicate that a natural restorative mechanism of sleep homeostasis begins to counteract the effects of prolonged wakefulness, potentially reducing the impact of sleep deprivation on the brain's phosphoproteome. Such a mechanism could be operating in our study, where sleep deprivation beyond a certain threshold (e.g., 9 hours) might trigger compensatory processes that prevent large-scale changes in the phosphoproteome.

Furthermore, while no significant changes were observed in individual phosphopeptides between the PS and AD groups, our analysis revealed that the whole-brain phosphorylation profile was still altered in the PS group (**Fig. 3e, f**). This suggests that while the differences between PS and AD groups were less pronounced compared to more traditional sleep deprivation methods, there was still a detectable shift in the brain's overall phosphorylation status. This highlights that the pro-survival (PS) group, despite its intermittent sleep, underwent some degree of cellular signaling changes that differentiate it from the AD group.

Lastly, the apparent minimal differences between the PS and AD groups could reflect a new function or regulation mechanism of sleep that is not fully captured by traditional methods. It has been suggested that the PONE (point of no return) state observed in the latter stages of prolonged sleep deprivation represents a critical shift in the ability of the brain to generate natural sleep (**Fig. 2e**). This loss of sleep potential, coupled with prolonged wakefulness, may signal a novel aspect of sleep regulation that warrants further exploration.

In conclusion, the small phosphoproteomic differences between the PS and AD groups likely stem from the intermittent nature of sleep during the Pr-SD phase, the buffering effects of sleep homeostasis mechanisms, and the fact that the SWAM model does not induce the same drastic changes as classical sleep deprivation protocols. These findings suggest that the brain may employ adaptive responses to moderate and prolonged sleep loss, and further research into the molecular mechanisms underlying these responses could provide deeper insights into the regulation of sleep and its impact on the phosphoproteome.

4. Sleep Deprivation, Sleep Need, and the PONE State

The paradox observed in PD mice—where increased sleep need (as indicated by SNIPPs) is coupled with a failure to sleep—poses an intriguing challenge to our understanding of sleep regulation during prolonged sleep deprivation. This contradiction could offer valuable insights into the complex mechanisms driving the progression of sleep deprivation and the transition to the PONE state. Our hypothesis proposes that this paradox may be explained by an intrinsic modulator, such as midbrain dopamine (DA) neurons, which decouples the relationship between high sleep pressure and the generation of sleep[3]. This decoupling mechanism would allow for greater flexibility in sleep regulation and prevent the abrupt cessation of sleep in the face of high sleep pressure, potentially underpinned by motivational and emotional factors[3].

Sleep Pressure and Motivational Drive

The two-process model of sleep regulation, which distinguishes between circadian (process C) and homeostatic (process S) components, has long served as the foundation for understanding sleep patterns[4]. While this model effectively explains many sleep-related phenomena, it does not account for all observed behaviors, particularly in extreme conditions of sleep deprivation. Across various species, researchers have noted adaptive sleep behaviors in response to environmental pressures, such as reduced sleep during bird migration[5] or the extended wakefulness seen in postpartum dolphins[6]. These adaptations, which occur without detrimental neurobehavioral effects, challenge the classical view of sleep need and its homeostatic regulation.

One possible explanation for these phenomena is the modulation of sleep by dopamine (DA), a key neurotransmitter involved in wakefulness and motivation[7]. Recent research by Karim and colleagues demonstrated that silencing midbrain DA neurons in mice induces a hypersomnia phenotype, with a modest reduction in the density of slow-wave activity during NREM sleep, while maintaining a normal homeostatic sleep response[3]. This suggests that DA plays a critical role in regulating sleep, potentially by providing the flexibility for adaptive sleeplessness under certain conditions, such as prolonged wakefulness.

We hypothesize that a similar mechanism may be at play in the PD mice observed in our study. Specifically, the decoupling of high sleep pressure from the ability to sleep could be mediated by DA or other intrinsic modulators in the brain. In situations of extreme sleep deprivation, such as during SWAM, this decoupling mechanism may become compulsively activated, overriding the normal drive for sleep and preventing the mice from entering sleep despite high sleep pressure. This mechanism could be an adaptive response that prevents sudden sleep onset under conditions of extreme situations, which would be incompatible with survival in the face of continuous waking.

Sleep Rebound and the Lack of Compensatory Sleep

Another puzzling aspect of our findings is the lack of sleep rebound in the RP4 group, which contrasts with the expected compensatory sleep following sleep deprivation according to sleep homeostasis theory. Traditionally, sleep rebound after deprivation is thought to reflect the body's effort to restore homeostasis and compensate for lost sleep. However, in our study, no substantial rebound sleep was observed in the RP4 group compared to baseline sleep levels, which requires further explanation.

One key difference between the SWAM protocol and classical sleep deprivation methods is that SWAM does not induce complete and immediate sleep loss. Instead, sleep is progressively reduced in a more fragmented manner, particularly during the early phases of deprivation (**Fig. 1e and Supplementary Fig. S1b**). This gradual increase in sleep loss, coupled with the forced wakefulness in a water environment, could be a critical factor in the absence of compensatory sleep rebound. In classical models of sleep deprivation, where complete sleep loss occurs within a short time frame (e.g., 6 hours), rebound sleep is more pronounced because the body is attempting to recover from a large sleep deficit. However, in the SWAM model, sleep is constantly interrupted, and only partial sleep deprivation occurs at any given time. The resulting sleep need accumulation is more gradual, and this may impair the brain's ability to generate and maintain sleep in a stable manner.

In disk-over-water studies, it has also been reported that, except for a slight increase in REM sleep (REMS), no obvious NREM sleep (NREMS) rebound occurred after several days of prolonged sleep deprivation (Pr-SD)[8]. This observation is consistent with the findings in our study, where no significant NREMS rebound was observed despite the accumulation of sleep need during the SWAM protocol.

Moreover, the unique environment in the SWAM model, which forces the mice to remain standing in the water, may further hinder sleep generation. The discomfort associated with maintaining an upright posture could significantly affect both the initiation and maintenance of sleep, even during the brief sleep bouts allowed during the early stages of SWAM. This disruption to sleep generation could persist into the later phases of the experiment, affecting the ability of the mice to rebound their sleep after the RP4 phase.

Although EEG recordings did not show a significant compensatory sleep rebound in the RP4 phase, the brain's phosphoproteomic changes were consistent with sleep homeostasis theory. These changes exhibited regular oscillation before and after RP4, suggesting that the brain was still attempting to restore its homeostatic balance, albeit in a manner that was not reflected in typical sleep patterns. This indicates that while traditional sleep rebound may not have occurred, there were still underlying physiological processes working to restore the brain's functional state in response to prolonged wakefulness.

5. Potential candidates involved in Pr-SD-induced lethality

In this study, we demonstrated the effects of Sik3 and PP2A as examples of kinases and phosphatases influencing Pr-SD-induced lethality. As revealed by our kinome and

phosphatome analysis of phosphoproteomic data, a significant proportion of kinase (63%) and phosphatase (60.8%) families exhibited changes in PD samples. This suggests that phosphorylation-based signaling pathways are broadly disrupted during Pr-SD, implying that multiple kinases and phosphatases may play a role in regulating Pr-SD-induced lethality. While our findings specifically highlighted the dual role of the S565A mutation in *Ppp2r5d* (PP2A) in both survival and sleep regulation, it is possible that other phosphatases, such as PP1 or PP2B, could have similar effects. Notably, recent studies have reported that the knockout of *Ppp3ca* and *Ppp3r1* (members of the PP2B family) influences sleep regulation[9], suggesting that PP2B phosphatase activity may also have the potential to affect survival under Pr-SD conditions. In future studies, targeted overexpression or knockout manipulations of genes known to regulate sleep could help identify additional genes that influence survival during Pr-SD. This approach may uncover a broader network of kinases and phosphatases involved in the molecular mechanisms underlying Pr-SD-induced lethality.

References

1 Wang, Z. et al. Quantitative phosphoproteomic analysis of the molecular substrates of sleep need. *Nature* **558**, 435-439 (2018).

2 Franken, P., Chollet, D. & Tafti, M. The homeostatic regulation of sleep need is under genetic control. *J. Neurosci.* **21**, 2610-2621 (2001).

3 Fifel, K., El Farissi, A., Cherasse, Y. & Yanagisawa, M. Motivational and Valence-Related Modulation of Sleep/Wake Behavior are Mediated by Midbrain Dopamine and Uncoupled from the Homeostatic and Circadian Processes. *Advanced science (Weinheim, Baden-Wurttemberg, Germany)* **9**, e2200640 (2022).

4 A.BORBELY, A. & ACHERMANN, P. Concepts and models of sleep regulation: an overview. (1992).

5 Rattenborg, N. C. et al. Evidence that birds sleep in mid-flight. *Nat. Commun.* **7**, 12468 (2016).

6 Lyamin, O., Pryaslova, J., Lance, V. & Siegel, J. Animal behaviour: continuous activity in cetaceans after birth. *Nature* **435**, 1177 (2005).

7 Kim, H. R. et al. A Unified Framework for Dopamine Signals across Timescales. *Cell* **183**, 1600-1616.e1625 (2020).

8 Everson, C. A. et al. Sleep deprivation in the rat: IX. Recovery. *Sleep* **12**, 60-67 (1989).

9 Wang, Y. et al. Postsynaptic competition between calcineurin and PKA regulates mammalian sleep–wake cycles. *Nature* (2024).
